# A Copernican Approach to Brain Advancement: The Paradigm of Allostatic Orchestration

**DOI:** 10.3389/fnhum.2019.00129

**Published:** 2019-04-26

**Authors:** Sung W. Lee

**Affiliations:** Scholarly Projects Unit, Department of Academic Affairs, University of Arizona College of Medicine—Phoenix, Phoenix, AZ, United States

**Keywords:** homeostasis, allostasis, evolution, blood pressure, posttraumatic stress disorder, complexity, criticality, neuroethics

## Abstract

There are two main paradigms for brain-related science, with different implications for brain-focused intervention or advancement. The paradigm of homeostasis (“stability through constancy,” Walter Cannon), originating from laboratory-based experimental physiology pioneered by Claude Bernard, shows that living systems tend to maintain system functionality in the direction of *constancy* (or similitude). The aim of physiology is to elucidate the factors that maintain homeostasis, and therapeutics aim to correct abnormal factor functions. The homeostasis paradigm does not formally recognize influences outside its controlled experimental frames and it is variable in its modeling of neural contributions. The paradigm of allostatic orchestration (PAO) extends the principle of allostasis (“stability through change”) as originally put forth by Peter Sterling. The PAO originates from an evolutionary perspective and recognizes that biological set points *change* in anticipation of *changing environments*. The brain is the organ of central command, orchestrating cross-system operations to support optimal behavior at the level of the whole organism. Alternative views of blood pressure regulation and posttraumatic stress disorder (PTSD) illustrate differences between the paradigms. For the PAO, complexities of top-down neural effects and environmental context are foundational (not to be “factored out”), and anticipatory regulation is the principle of their interface. The *allostatic state* represents the integrated totality of brain-body interactions. Health itself is an allostatic state of *optimal anticipatory oscillation*, hypothesized to relate to the state of criticality, a mathematical point of poise between phases, on the border between order and disorder (or the “edge of chaos”). Diseases are allostatic states of impaired anticipatory oscillations, demonstrated as rigidifications of set points across the brain and body (disease comorbidity). Conciliation of the paradigms is possible, with “reactive homeostasis” resolved as an illusion stemming from the anticipation of environmental monotony. Considerations are presented with respect to implications of the two paradigms for brain-focused intervention or advancement; the hypothesis that the state of criticality is a vehicle for evolutionary processes; concordance with a philosophy of freedom based on ethical individualism as well as self-creativity, non-obsolescence, empowerment, and citizenship; and concluding reflections on the science and ethics of the placebo, and the potential for virtuous cycles of brain-Anthropocene interactions.

## Introduction and Overview

The human brain is the most precious resource on our planet. Excitement about the brain takes many forms, and actions on behalf of the brain go in many different directions. It may be valuable to articulate and clarify whether there are particular approaches to the brain that favor the recognition of particular categories of data, and whether they lead to different goals and strategies for intervention. Such an exercise may help visualize alternative trajectories for brain-related advancement at the level of “broad brush strokes.”

This essay contends that there are two main paradigms (Kuhn, 1962) for brain science and brain-focused intervention. The earlier views the brain as one organ among others, a collection of cells and tissues that regulates itself on the basis of corrective feedback. The latter views the brain as the seat of central command for the organism as a whole, and one that operates on the basis of anticipatory regulation. The current dominance of the earlier paradigm tends to obscure recognition that its associated explanations entail the cropping of large swaths of data, which are central to the latter. From the point of view of biological theory, the core difference between the paradigms derives from the latter’s evolutionary perspective, which entails greater recognition of the relationships between the brain and the body, and between the brain and the natural environment. The objective of this presentation is to explore historical, scientific, interventional, and other differences between the two paradigms, so that innovators, researchers, practitioners, policy-makers, patients, end-users, and others can gain clarity with respect to both the explicit and implicit assumptions associated with brain advancement agendas of any kind.

Over the course of three decades, a series of brain-centric, evolution-inspired insights have been articulated with increasing refinement, as principles of *allostasis* (Sterling and Eyer, 1988; Sterling, 2004, 2012, 2014). Allostasis recognizes that the role of the brain is to serve as the integrative center for anticipatory regulation, to orchestrate operations across systems, and thereby support behavioral optimality for successful interaction with the environment at large. Because ensuing usage of the term “allostasis” by other investigators has sometimes not recognized the full significance of the brain-centric and cross-system perspective first put forth by Sterling, this article refers to the *paradigm of allostatic orchestration* (PAO), to point toward his original ideas and to extend them.

The essay reviews the origins and principles of homeostasis and allostasis and illustrates their differences by considering how they approach the phenomena of blood pressure regulation and posttraumatic stress disorder (PTSD). It contends that the Sterling principle of allostasis is sufficiently different that it warrants characterization as a new paradigm—the PAO—which is articulated as a set of propositions, and it also contends that the two paradigms can be conciliated. Selected considerations are presented pertaining to their different interventional implications; a falsifiable model regarding the vehicle or mechanism of evolutionary processes; the alignment of the PAO with a philosophy of freedom; and the proposed utility of allostatic planning and action in the context of the Anthropocene epoch.

## The Paradigm of Homeostasis

### Origins of Homeostasis

The career of Claude Bernard, generally considered to be the father of modern experimental physiology, encompassed seminal discoveries including the role of the pancreas in digestion and the glycogenic function of the liver. He conducted numerous studies showing the influence of the brain and nervous system on digestive processes, and in 1858 he reported neural regulation of blood vessel dilation. Yet it was his experimental methodology itself that proved to be his greatest legacy. As a pioneer for the scientific revolution and the role of the laboratory, Bernard brought reductive physico-chemical observation and reasoning to the study of living organisms, even as he approached his work with reliance on a form of intuition that led him to be critical of strict positivism or doctrinaire materialism (Grmek, 2008). Though not a “vitalist,” Bernard “attributed a ‘directive and creative’ idea to life” which also necessitated the use of vivisectional techniques for the revelation of findings not demonstrable otherwise. Eventually he came to categorize life into three different forms, and of the third category—higher vertebrates—he proclaimed that only they were truly “free,” in that they alone possessed the complex mechanisms which permitted them to maintain the fixity of physical conditions (temperature, nutritional substrates, acidity, etc.)—the *milieu interieur* (Bernard, 1878)—that is necessary for life.

Historians typically credit Walter Cannon for being the giant who received the kernel of Bernard’s concept of the *milieu interieur*, and amplified it as the concept of *homeostasis* (Cooper, 2008). As an experimentalist, he was among the first to use roentgenography to characterize the workings of the digestive system. Critically, he observed that when animals were emotionally excited there was a cessation of gastric and intestinal function, and he went on to establish that the sympathetic nervous system was deployed under “emergency” circumstances. During World War I he conducted research on the causes of acidosis after traumatic shock, and subsequently he developed numerous techniques for investigation of the autonomic nervous system including the chemical transmission of neural impulses (Benison and Barger, 2008).

Potentially, it may have been his studies of sugar mobilization in the setting of insulin-induced hypoglycemia, that may have spurred his attention to the organizing principle of *homeostasis*. Cannon credited Bernard as being the first to give a “precise analysis” to the general idea that living beings have a special capacity to maintain their stability in the face of the changing environment, and then refined his own understanding as the following set of postulates (1929):

1. In an open system such as our bodies represent, compounded of unstable material and subjected continually to disturbing conditions, constancy is in itself evidence that agencies are acting, or ready to act, to maintain this constancy.
2. If a state remains steady it does so because any tendency towards change is automatically met by increased effectiveness of the factor or factors which resist the change.
3. Any factor which operates to maintain a steady state by action in one direction does not also act at the same point in the opposite direction.
4. Homeostatic agents, antagonistic in one region of the body, may be cooperative in another region.
5. The regulating system which determines a homeostatic state may comprise a number of cooperating factors brought into action at the same time or successively.
6. When a factor is known which can shift a homeostatic state in one direction, it is reasonable to look for automatic control of that factor or for an opposite factor or factors having an opposite effect.

In concordance with these postulates, laboratory physiological research from the early twentieth century and beyond has made tremendous progress in the understanding of *factors* which move system parameters in one direction or another.

### Examples of Homeostasis

#### Actors and Anomalies in the Homeostatic Understanding of Blood Pressure Regulation

Blood pressure regulation is a phenomenon exhaustively studied under the homeostatic paradigm. The main actors for blood pressure maintenance include the heart, which acts as a pump; the vessels, which constrict and relax as they transport blood throughout the body; and the kidneys, which manage blood volume by filtering fluid and conserving sodium to preserve osmotic pressures. Exhaustive search for the factors of blood pressure regulation and their sites of production or action has led to recognition that the adrenal glands, lungs, and liver also play roles. The main “equation” renders blood pressure to be a function of cardiac output and total vascular resistance, analogous to Ohm’s law in physics where the voltage in an electrical circuit is calculated as the product of current and resistance. Cardiac output is itself a product of heart rate and stroke volume, with stroke volume, in turn, being influenced by blood volume (“preload”), the pressure against which the heart must contract (“afterload”), and the strength of each contraction (contractility). If sensors in the carotid artery or kidney detect a decrease in blood pressure, there will be a cascade of factor actions that alter volume, vascular resistance, and cardiac output, to increase it. If they detect a rise in blood pressure, there will be cascades in the opposite direction. Life itself—through the capacity for homeostasis—is given the credit for checks and balances that preserve the *normal* level of blood pressure.

When the system fails to maintain its normal state—for example hypertension, the state of persistent blood pressure elevation—then causes are sought in the various factors which would otherwise return the blood pressure to “normal.” Yet though there are certain well-characterized abnormalities of homeostatic mechanisms, that are correctable causes of hypertension—for example renal artery stenosis or endocrine tumors—many anomalies arise when explaining blood pressure regulation as the steady state of local tissue factors. Ninty-five percentage of individuals with hypertension are deemed to have a type that has no identifiable local factor as the cause (Carretero and Oparil, 2000). Among them are those with “white coat” hypertension, defined as blood pressure that is elevated when measured in a clinic setting, and not elevated when measured outside the clinic (Pickering et al., 1988). Nor can local factors explain why some forms of sudden psychological disruption should be associated with an immediate and sharp drop of blood pressure (that can be extreme enough to produce loss of consciousness, i.e., vasovagal syncope), not corrected by an immediate rise. Furthermore a therapeutic anomaly of the homeostasis paradigm is shown in evidence which indicates that “mental” (non-physical) interventions, that by definition do not “directly” interact with any known physical factors, can nonetheless have an impact on blood pressure (albeit to modest degrees, e.g., Bai et al., 2015). Despite inordinate study of blood pressure homeostasis, the complexity of factor interactions (or the difficulty of accommodating anomalies) is reflected in the absence of a consistent schematic across textbooks of physiology or medicine, to represent a broad scientific consensus regarding its essential mechanisms.

#### Progress and Limits in the Homeostatic Understanding of PTSD

PTSD is commonly conceptualized as being a “psychological” condition, one that may ensue following the experience of a *non*-physical trauma (e.g., an emotional shock, such as being witness to a violent event). Thus, studies which aim to explore *physical* changes in PTSD are themselves conducted in some defiance of reductive explanation based on *physical* factors alone. Nonetheless, stimuli can be carefully designed with the intention of producing controlled forms of trauma within a laboratory setting, and the brain can be taken to be the organ of behavior (akin to the heart, vascular system and kidneys being the organs of blood pressure). By refining such frameworks for experimentation, specific physical factors for PTSD have been sought in cellular failures to extinguish associative conditioning between environmental cues and fear learning mechanisms (VanElzakker et al., 2014), and studies of individuals with PTSD (or those at increased risk for it) have demonstrated alterations in brain circuits for processing and modulation of the emotions, a variety of dysregulations in neurohormonal activity patterns, and genetic and other differences compared to control populations (Yehuda et al., 2015). Laboratory investigation has led to a dramatic expansion in the understanding of brain functionality beyond the condition of PTSD, as such studies have valuably elucidated much of the neural mechanics of our far-reaching arousal and stress response systems.

Yet a slew of anomalies arises when understanding for PTSD depends on a reductionist focus on any single factor, category of variable, or level of analysis or intervention. In the first place, fundamental complexity pertaining to “PTSD” is evidenced by the observation that among some military populations the condition is not even fully recognized as a “disease” (Fisher, 2014), and instead, its phenomenology is conceptualized as a “normal” feature of the vocation. In some cases, a traumatic event may lead to an alternative and “positive” trajectory of posttraumatic growth (Tedeschi and Calhoun, 2004), which may depend significantly on cognitive processing that has not been shown to be a determined outcome of neural processes. In another vein related to cognitive appraisal, PTSD symptom severity may be associated with veterans’ perception of their homecoming reception (Johnson et al., 1997). Yet at the other pole of perception for the “physical reality” of PTSD, patients diagnosed with the condition have increased risk for organ system impacts including cardiovascular disease (Beristianos et al., 2016), metabolic disturbances (Farr et al., 2015), and accelerated physiological aging (Williamson et al., 2015). In the realm of therapeutics, a recent review lamented that “…at a time when there are more biological findings in PTSD than in almost any other psychiatric disorder, there are no drug targets” (McFarlane et al., 2017). The relative lack of traction is further shown in the limitations of even “evidence-based” forms of trauma-specific cognitive therapies for PTSD (Steenkamp et al., 2015), which have high drop-out rates (Najavits, 2015) and minimal impact on the fundamental problem of sleep disturbance (Pruiksma et al., 2016).

## The Sterling-Eyer Principle of Allostasis

### Origins of Allostasis

Through the mid-nineteenth century, geologists and naturalists increasingly debated whether life forms on earth had all existed throughout time, or whether they had evolved from one form into another. For those who held an evolutionary perspective, the mechanism of change had remained a mystery. In 1858, the same year that Claude Bernard discovered neural regulation of the blood vessels (and one year after Bernard introduced the concept of the “milieu interieur”), the naturalist Alfred Russel Wallace wrote a letter to Charles Darwin in which he proposed that species may change through the pressures of scarcity generated by nature itself, which would lead to the differential reproductive success of some variants over others. Subsequently, Darwin (1859) published *On the Origins of Species by Means of Natural Selection, or, the Preservation of the Favoured Races in the Struggle for Life*, and the influence of evolutionary thinking on subsequent science, culture, religion, and governance, can hardly be overestimated. By placing us in a lineage with other primates, Darwin’s writings stand out as the second decisive science-based humbling for the human species. At the same time, the vastly expanded perspective entailed by evolutionary thinking has encouraged a cosmological context for the human imagination.

The neuroscientist Peter Sterling has given the following account (2004) of the origins of *allostasis*, beginning with a personal appreciation for the influence of environmental context on biological regulation. In the 1960s, Sterling was working as both a neuroscientist and a social activist, and while canvassing in African-American communities he noticed that many individuals had limps or facial droops indicative of a history of stroke, as a consequence of the high prevalence of hypertension. Over the next two decades, Sterling integrated his naturalistic observation, which was being confirmed in epidemiological studies of the links between social disruption and chronic disease, with other growing bodies of knowledge. There were electron microscopic findings of the ubiquity of nerve fibers on blood vessels, endocrine cells, and elsewhere, suggesting that “the brain has close access to essentially every somatic cell,” and also primate studies showing that demands for sustained attention caused elevations of blood pressure and release of catabolic hormones.

Eventually Sterling and Eyer synthesized these insights as the principle of *allostasis* (1988), which states that “an organism must vary all the parameters of its internal milieu and match them appropriately to environmental demands.” If an individual is exposed to an environmental condition presenting potential danger and high need for vigilance, the brain orchestrates systems on an anticipatory basis, toward a physiological arousal state that is a match for those demands (Sterling and Eyer, 1988). For a context of social disruption, characterized by the persistence of perceived danger, the brain’s natural behavior is to produce a sustained state of high physiological arousal. Thus, whereas homeostasis models the cause of hypertension by pointing to abnormal functionality of the heart, blood vessels, kidneys or molecular signaling, allostasis explains that the brain is deliberately directing these organs on an anticipatory basis, in concert and with respect to context, to produce an elevation of blood pressure for delivery of resources (glucose, oxygen) to the large muscle groups.

### Allostasis Resolves Anomalies in the Homeostatic Model of Blood Pressure Regulation

Under allostasis, the phenomena of essential hypertension, white coat hypertension, vasovagal hypotension, and meditation-induced reductions of blood pressure are no longer anomalous. They are all due to the transduction of influences—chronic or acute stressors, other environmental variables, or mental training—from the brain to the cardiovascular system. Many (not all) homeostatic schematics of blood pressure regulation do highlight the brainstem centers of the autonomic nervous system; direct neural control and afferent signaling between the brain, heart and blood vessels; and neural ganglia at the kidneys. Nonetheless, the role of neocortical influences on the brainstem and autonomic signaling has yet to be understood in depth, and the full significance of these pathways for neural influence has yet to be appreciated across physiological subspecialties, clinical medicine or public health.

For example, there is now an expansive corpus of studies that have provided an increasingly granular appreciation for the environmental factors associated with blood pressure, including an early report from Bevan et al. (1969). In groups of normotensive, hypertensive, and borderline hypertensive individuals, blood pressure has been shown to be higher in work environments compared to home or during sleep, for all three groups (Pickering et al., 1982). The concept of “masked hypertension” (Pickering et al., 2002) points to individuals who show elevated blood pressure outside the clinic setting—that is likely to be of pathological significance—with lower-range readings at the clinic. Ambulatory and home-based readings show that blood pressure is higher in the winter (Sega et al., 1998), and furthermore weather-dependent variations in blood pressure appear to be explained by a synoptic measure of air mass, and not by differences in temperature, cloud cover, relative humidity, atmospheric pressure, or wind speed on a univariate basis (Morabito et al., 2008).

The brain-centric allostatic perspective in conjunction with the polyvagal theory (Porges, 2007) can also explain the existence of both hypertensive and hypotensive responses to an acute psychological stressor (fighting vs. fainting). The existence of variability for production of these starkly different response directionalities may reflect a hierarchical organization of autonomic response patterns that derive from evolutionary phylogeny—the fight or flight response characteristic of mammals produces more behavioral options (at a higher metabolic cost), compared to the “freeze” mode more dominant in reptiles. With respect to a potential mechanism, it has been contended that likelihood to engage a fight vs. a freeze response may be influenced by variability in individual traumatic stress histories, which are “imprinted” as differential patterns of activity at the level of the right and left cerebral hemispheres, and especially the insular cortex (Lee et al., 2014).

Too, the Sterling principle of allostasis may shed light on the controversy around the role of salt consumption as a factor in hypertension. The allostasis perspective is consistent with the idea that dietary salt may be not a culprit but rather an “innocent bystander” (DiNicolantonio et al., 2017). It is the brain itself, under stress, that establishes an elevated set point for blood pressure, and a variety of physiological and behavioral mechanisms may be recruited accordingly—including a propensity for salt consumption, in order to maintain a higher circulatory volume. Thus, while low-salt diet interventions for hypertension management may lead to short-term reductions of blood pressure, they also appear to result in increased levels of renin, aldosterone, and adrenergic tone (Graudal et al., 2017)—driven by the brain, to compensate for the relative volume depletion. This explanatory framework adds weight to the unanswered question of whether a low-salt diet has benefits—or even harms—as a strategy for cardiovascular adverse event prevention (Braam et al., 2017).

## The Paradigm of Allostatic Orchestration (PAO)

### Allostasis—From Principles to Paradigm

In his chapter-length exposition (2004), Sterling articulated allostasis as the following set of principles: (1) organisms are designed for efficiency; (2) efficiency requires reciprocal trade-offs (managed by the brain); (3) efficiency requires predicting (by the brain) what will be needed; (4) prediction requires each sensor to adapt its sensitivity to the expected range of input; (5) prediction requires each effector to adapt its output to the expected range of demand; and (6) predictive regulation depends on behavior whose neural mechanisms also adapt.

An objective of the present essay, addressed in greater depth in Section “Conciliation of the Two Paradigms,” is to help resolve questions and occasional debate as to whether the concepts or principles of allostasis merit the cognitive costs that come with the introduction of new language. Post-Sterling, many have used the allostasis concept to refer to the general phenomenon that biological set points become temporarily altered under the condition of acute environmental stress (and also in order to point toward the concept of allostatic load, reviewed in Section “Propositions of the PAO”). Yet Cannon himself recognized that systems do not maintain absolute constancy in their set points, and he was careful to note that he had chosen the prefix “homeo” (similar) rather than “homo” (same) when coining the term homeostasis (1929). It seems likely that the answer to the question, whether or not allostasis is simply homeostasis in “new clothes,” lies mainly in Sterling’s heightened appreciation for the brain. Sterling’s recognition that the brain is necessary as a dedicated organ for higher-order processing, for management of trade-offs or anticipatory orchestration among actors, is in some respects the same conclusion reached by Bernard more than 100 years earlier.

**Figure 1:**
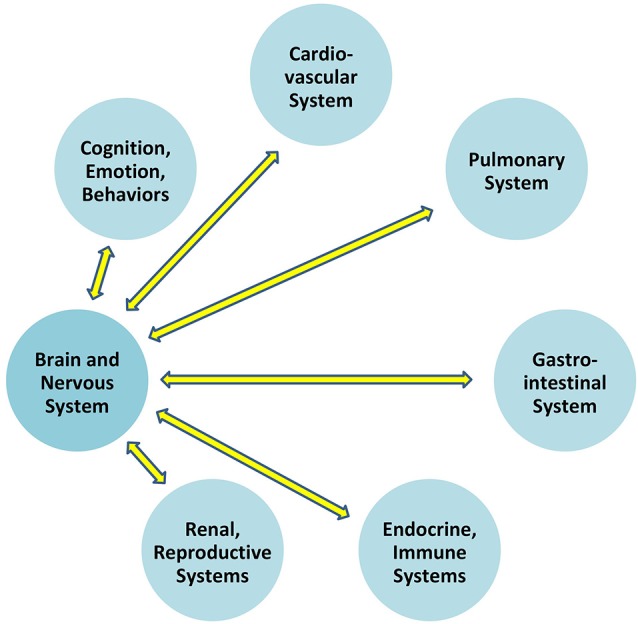
Bidirectional communication exists between the brain and all other organ systems.

**Figure 2:**
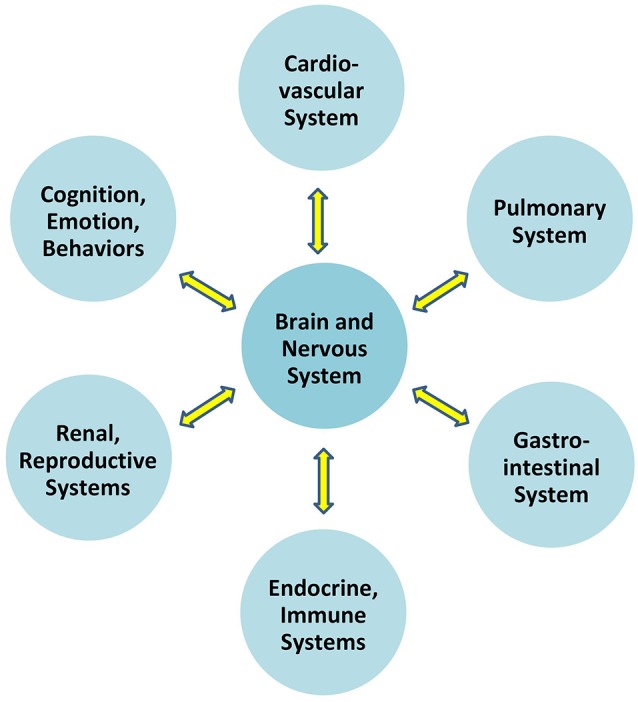
Overall brain-body relationships as viewed under the paradigm of allostatic orchestration (PAO). As the organ of central command, the brain orchestrates operations across systems, for anticipatory regulation of whole-organism behaviors.

Communication which permits the brain to regulate blood pressure is at play *between the brain and all organ systems* (Figure 1). In particular, the autonomic nervous system exerts constant, distributed, and calibrated “accelerator and brake” effects, through the sympathetic and parasympathetic divisions respectively, to orchestrate regulation *across* organ systems, and for precision-guided environmental engagement (Rees, 2014). Gross anatomy shows direct autonomic innervation to the major internal organs, and immune cells too are directly responsive to parasympathetic signaling (Pavlov and Tracey, 2015). The summed influences of autonomic, neuroendocrine, and other brain-directed mechanisms including synchronization between the “master clock” in the suprachiasmatic nucleus and peripheral clocks throughout the body (Dibner et al., 2010), call forth Bernard’s statement (Conti, 2002) that bi-directional communication between the brain and other systems is the critical organizing principle of human biology (Figure 2).

Ultimately there are three main reasons to warrant the demarcation of allostasis as a new paradigm (Kuhn, 1962). Allostasis explicitly models that parameters will vary for optimizing interaction with the changing and complex variables of the natural environment, whereas homeostasis looks for the factors that maintain parameter constancy, typically within the context of a controlled laboratory setting; it explains that successful biological regulation takes place on an anticipatory basis, whereas homeostasis generally explains biological regulation on the basis of corrective or reactive feedback; and allostasis points to the role of the brain as the organ of central command, whereas homeostasis may or may not include the complexities of neural regulation as formal variables within the experimental frame. As emphasized throughout this essay, these distinctions point to different categories of data of interest, different model problems, and different model solutions.

### Propositions of the PAO

**Figure 3:**
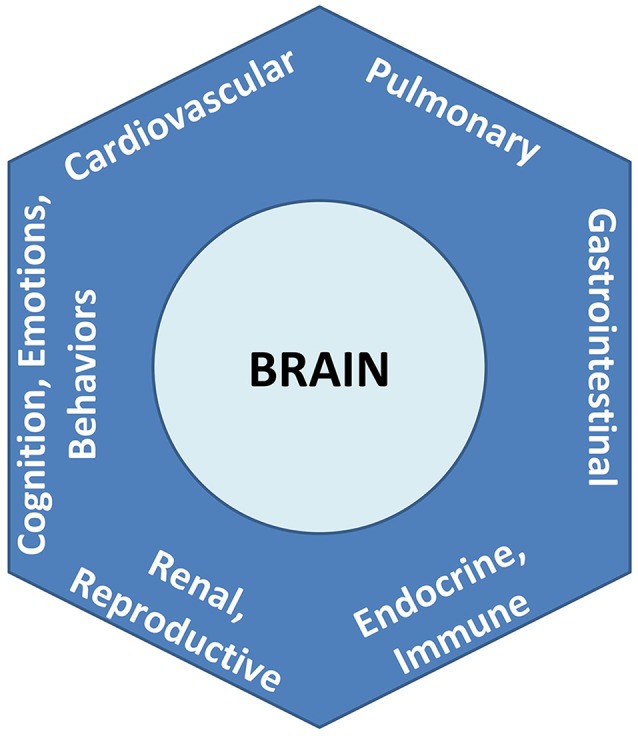
Allostatic state, representing the totality of integrated brain-body interactions. Each system’s behavior is a *facet* of the unitary state.

To formalize its recognition that brain-body relationships are the rule and not the exception, the PAO applies the nomenclature of an *allostatic state* to refer to the integrated totality of interactions between the brain and all organ systems (Figure 3). Notably and as explored further below and in Section “Conciliation of the Two Paradigms,” this definition for an “allostatic state” differs from that given by other investigators (e.g., Koob and Le Moal, 2001, and see Table 1). The expression of neurally-regulated functioning within a given system can be conceptualized as a *facet* of an allostatic state. White coat hypertension (Pickering et al., 1988), for example, may be understood as the cardiovascular facet of the allostatic state of anxiety, among selected individuals when they are in the presence of a medical authority figure. Other examples of findings or symptoms associated with allostatic state facets, that are typically associated with environmental, social, or perceived stressors, include irritable bowel syndrome (e.g., Mayer et al., 2001), reproductive disturbance (e.g., Rooney and Domar, 2018), bronchospasm (e.g., Rosenberg et al., 2014), immune dysregulation (Glaser and Kiecolt-Glaser, 2005), chronic pain (e.g., Burke et al., 2017), and addictive behaviors (e.g., Koob and Schulkin, 2018). Importantly, the PAO’s conception of the allostatic state does not apply to pathology alone, nor does it imply that neural effects (or stressors) are the sole cause of any disorder; rather it serves to remind that there is always bidirectional influence between any given system expression and the functioning of the brain.

**Table 1:** Different meanings for concepts associated with homeostasis, allostasis, and the PAO.

Term	Cannon (1929) and followers	Sterling (2004, 2012)	McEwen and followers (McEwen and Wingfield, 2003)	Proposed new usage under homeostasis revised, without allostasis (see text)	Proposed usage under the PAO
Homeostasis	The *relatively* *constant* state of a parameter, *despite* changing environmental conditions	Generally same as Cannon; needs replacement because constancy is not demonstrated when there is systematic attention to environmental and top-down neural influences	Same as Cannon, with a focus on a few central parameters including core temperature, pH, glucose, oxygen tension	Same as Cannon, however with major conceptual, procedural, empirical, and inferential revisions, caveats, etc (potentially, “kludges”)	Generally same as Cannon; appreciated as a simplified model that does not recognize critical complexities; known to incur risks of producing misleading inferences
Homeostatic mechanisms	Interactions of numerous and various factors, across systems, inspiring search for molecular targets of therapy	Generally same as Cannon; and with critical awareness of the absence of attention toward systematic environmental and top-down neural influences	Mechanisms that preserve temperature, pH, glucose, oxygen tension	Same as Cannon; expanded for more systematic recognition of reactive vs. predictive types, environmental and top-down neural influences	Generally same as Cannon and Sterling; same caveats as above
Allostasis	n/a	Systematic variation in parameters at the direction of the brain (as prediction-machine), based on anticipatory regulation, to aid whole-organism evolutionary objectives	Interactions of numerous and various factors that support homeostasis under acute stress, and also on an anticipatory basis in nature	n/a	Per Sterling (without dependence on natural selection); adjective *allostatic* used as “information-dense” shorthand to refer to environmental influences, top-down neural effects, and anticipatory regulation
Allostatic state	n/a	n/a	Chronic deviation from homeostasis due to psychosocial or environmental stress (Koob and Le Moal, 2001)	n/a	Integrated totality of brain-body interactions (irrespective of environmental factors); Koob-Le Moal conception potentially re-labeled as *allostatic strain*
Allostatic load, or allostatic impairment	n/a	n/a	Tissue damage from chronic exposure to allostatic mediators	n/a	Allostatic load is one example of an impaired allostatic state (or simply, allostatic impairment)
Allostatic orchestration	n/a	n/a	n/a	n/a	Synonym for “allostasis”, to highlight top-down neural and cross-system effects

*Definitions or explanations are paraphrased or presented with selective emphasis, in order to convey the essential similarities or differences compared to other authors*.

A second new concept of the PAO is *optimal anticipatory oscillation*, introduced as a way to help spark new thinking and scientific imagination for the operationalization of health. The concept is effectively the same as Sterling’s definition of health as “optimal predictive fluctuation” (2004), which can replace the idea that health is a capacity for reactive (or corrective) feedback. Optimal anticipatory oscillation builds on appreciation of the brain as a “prediction machine” (e.g., Engel et al., 2001; Buzsaki, 2006), and reflects the capacity for matching between operations in the individual with the typically oscillatory features of the environment, in ways that reduce limitations, enhance resilience, or expand the range of possible functionalities or opportunities. In the cardiovascular facet, for example, optimal anticipatory oscillation may be demonstrated as patterns of variability in heart rate, indicating a capacity for rapid recalibrations of cardiac output in context-sensitive ways, that confers a decreased risk of morbidity or mortality (Kleiger et al., 1987; Dekker et al., 1997). The behavioral facet of optimal anticipatory oscillation may be associated with characteristic sleep patterns (Buysse, 2014), motor behaviors or sensory acuity (McClintock et al., 2016), or positive cognitive appraisals (e.g., Kalisch et al., 2015). Optimal anticipatory oscillation to affect the facet of immunity is shown in a study of meditation combined with breath training and cold temperature exposure (Kox et al., 2014), that entailed the anticipatory tuning of cognitive appraisals and breathing cycles. Characterization of the allostatic state facets of optimal anticipatory oscillation—positive health across organ systems, to reduce the prevalence and incidence of morbidity, and confer resilience—is a ripe area for future research. In a sense, the agenda aims to bring tractability to the definition for health—a “state of complete physical, mental, and social well-being, and not merely the absence of disease”—given by the World Health Organization (1948) after World War II.

Integration of the above concepts permits a formal way to recognize and elucidate heterogeneity in phenotypes of health or disease. The PAO postulates a range of allostatic states, characterized by different tendencies for demonstrating optimal anticipatory oscillation. At one end, allostatic states of *optimality* indicate a dynamic capacity for recalibration that is generally associated with positive mental and physical health. At the other end, allostatic states of *impairment* indicate rigidification in operational set points, and they manifest as conditions of behavioral and physical disease. Between optimality and impairment, there is a spectrum of states associated with varying kinds and degrees of functionality. The multiplicity of disturbances represented in allostatic state facets is a prediction confirmed in the empirical finding that comorbidity (the presence of more than one disorder) is the rule among individuals with chronic diseases (Wolff et al., 2002).

**Figure 4:**
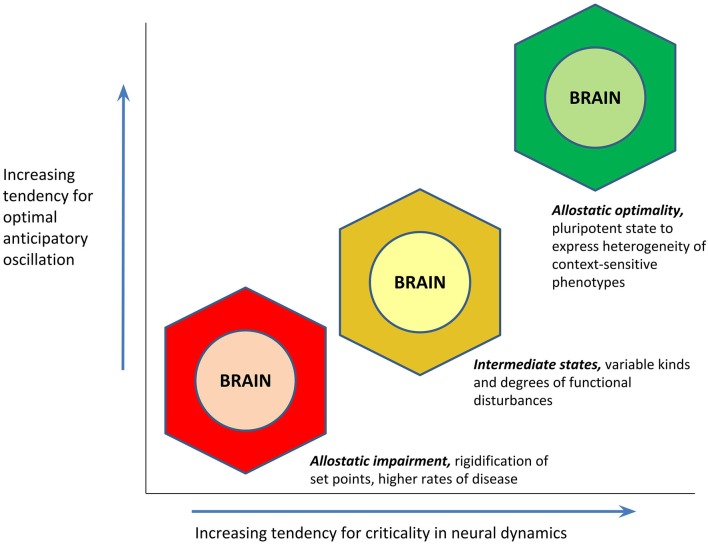
Hypothesized relationship between an allostatic state’s proximity to the state of criticality (mathematical balance, between phases, on the border between order and disorder), and its tendency to demonstrate optimal anticipatory oscillation. The function is proposed to be non-linear in concordance with the likely presence of power laws.

If optimal anticipatory oscillation is a way to operationalize health, the question still remains as to what might be the *cause* for this capacity. Whereas homeostasis loosely conceptualizes the cause of health as an “absence of abnormality in the factors of homeostasis,” the PAO’s attention to anticipatory regulation invokes as the cause of health that state of the brain (being the site of central command) that is most conducive to the generation of rapid and context-sensitive variations in its activity. A given brain activity pattern can only be characterized as “optimal” for a given environmental condition; *it is the pluripotent capacity to express a range of activity patterns, that should be the most optimal pattern of all*. An “empty” state of latent potentiality may have great import for anticipatory regulation, when external circumstances entail unpredictable and last-moment changes (up to and including “black swan” events; Taleb, 2007); such a state would serve as an “information reservoir” for yielding a repertoire of different behaviors, including entirely new ones. A hypothesis of the PAO is that increasingly optimal anticipatory oscillation—or the capacity for expressing context-sensitive and heterogeneous phenotypes of “health”—is a function of the brain’s proximity to the state of *criticality* (Beggs and Timme, 2012; Shew and Plenz, 2013; Cocchi et al., 2017). “Criticality” (in statistical physics, and in the PAO) refers to the point of mathematical balance between phases of a complex system, or a state of poise on the border between order and disorder, also described as the “edge of chaos.” Cortical functioning at criticality appears to permit optimizations of dynamic range (ability to respond to stimuli that are of many different sizes), fidelity of information transmission, and information capacity (Shew and Plenz, 2013). The potential relationship between cortical criticality and optimal anticipatory oscillation is presented in Figure 4. The theorized value of allostatic state criticality is further highlighted through depiction that the relationship between criticality and optimal anticipatory oscillation may be non-linear, given that as with other complex systems, many functions of the brain are likely to operate on the basis of power laws (e.g., Thiel, 2014; Bettinger, 2017; Kasagi et al., 2017).

As to the genesis and perpetuation of different states, the PAO hypothesizes that downward drift toward allostatic impairment is likely to be associated with chronic stress or trauma. *Allostatic load*, which is used to describe the condition where acutely valuable stress mediators have crossed a threshold—because of stress chronicity—leading to the production of tissue damage (McEwen and Stellar, 1993; McEwen, 1998), can be understood as one type of allostatic impairment. Long-term exposure to catabolic hormones and other stress-related mediators (glucocorticoids, sympathetic neurotransmitters, inflammatory cytokines) may lead to hypertension, hyperlipidemia, glucose intolerance, visceral obesity, and other diseases of “chronic stress” (Seeman et al., 2010). A midway point in this process, when stress has been chronic if not yet established as “full-blown” allostatic load, has been labeled as the “allostatic state” by Koob and Le Moal (2001), and it seems tenable that the Koob-Le Moal conception might instead be given the label of allostatic *strain*. Allostatic impairment may also occur on time scales longer than individual life spans, in that for example, many chronic diseases can be attributed to the persistence of evolutionarily ancient biological pathways (Lee et al., 2004; Yun et al., 2004; Goldman, 2015). While vasoconstriction, inflammation and thrombosis are critical responses for physical traumas that penetrate beyond the epidermis and thereby produce risks for exsanguination and potentially pathogenic microbe exposure, these same pathways are also likely to produce “diseases of modernity” when they are repeatedly activated as a consequence of perceived, social or other “non-physical” stressors (Sapolsky, 2004). In the other direction, elucidation of mechanisms for upward drift (toward allostatic optimality) is a bright area for future research, at both individual and population or species levels.

To summarize the preceding ideas, propositions of the PAO are presented as follows.

1. *Change itself is the norm of nature, and the natural environment is complex*. Realistic modeling to explain whole-organism behavior requires systematic attention to environmental complexity.
2. *The complexity of the brain permits optimized forms of engagement with environmental complexity, including anticipatory regulation*. The brain is a complex organ that serves as central command for the organism as a whole, to permit context-sensitive orchestrations of cross-system operations and parameter variations.
3. *Brain-body communication is the rule, not the exception*. Physiological mechanisms can be modeled as the integrated totality of brain-body interactions, or the allostatic state, with different system functionalities being explicable as different facets of the overall state.
4. *Healthiness and diseases can be conceptualized non-categorically, as a heterogeneity of phenotypes that exist along a continuum between allostatic optimality and impairment*. Health itself can be defined as optimal anticipatory oscillation, which refers to flexible and successful interactions with the complex and changing environment; diseases can be defined as the rigid persistence of context-insensitive operational set points.
5. *Healthiness, disease, and other demonstrations of anticipatory oscillation (or its impairment) are themselves secondary phenomena, which invoke the need for investigation of primary causes*. Tendency to maintain the state of criticality (a point of mathematical poise between phases, on the border between order and disorder) is hypothesized to explain the likelihood to express optimal anticipatory oscillations.

### Conciliation of the Two Paradigms

As noted in Section “Allostasis—From Principles to Paradigm,” the current presentation aims to help resolve the question of whether allostasis (to include the PAO) is genuinely different from homeostasis. Attention to this question entails empirical, analytical, inferential, interventional, and ethical considerations, some of which are further explored in Section “Selected Considerations and Summary.” Clarification of terminology shared by the two paradigms, as put forth by different authors, is presented in Table 1. A listing of selected properties of both paradigms, to include ways that the PAO differs from yet can be conciliated with homeostasis, is contained in Table 2.

**Table 2:** Selected properties of the paradigm of homeostasis and the paradigm of allostatic orchestration (PAO).

	Homeostasis	PAO
Core concept	Stability through constancy (or similitude)	Stability through change (resembles “constancy” when product of neural and environmental complexities is minimized)
Emergence in the history of biology	Originally motivated to apply nineteenth century physico-chemical insights toward reductionistic understanding of life processes; procedures of the laboratory established prior to Darwin	Motivated by naturalistic observations including the influence of large-scale environmental variables, as well as the “prediction-machine” integrative role of the brain for navigation of complex and changing environments; informed by a post-Darwinian evolutionary perspective; principles of physics and chemistry still apply
Experimental frame	Laboratory, controlled conditions	Open contexts and changing conditions, which may mimic the laboratory under rare circumstances
Primary variables of interest, and regulatory model	Genes and molecules; interactions occur on the basis of corrective feedback	Parameters of complexity pertaining to neural function and the environmental context, as well as mediator pathways; interactions occur on the basis of anticipatory behavior
Principle mode of causal inference	Molecular interactions determine phenomena at higher levels of observation (“bottom up” explanations)	Phenomena at higher levels of observation including neocortical activity and environmental factors may cause or correlate with phenomena at lower levels of observation (explanation based on “top-down” or bidirectional influences)
Status of the brain	One organ among others	One organ among others, yet also the central and integrative site of information processing for all organs and behaviors
Definition of health	Absence of disease	Optimal anticipatory oscillation for complex and changing environments (context-sensitive phenotypic heterogeneity), theorized to be a function of system criticality
Explanation for pathology	Production of categorically dysfunctional molecular interactions	Persistence of molecular interactions that are not beneficial for a given context, in association with neurally-directed rigidification of oscillatory patterns
View of disease comorbidity	Not emphasized, due to focus on system-specific mechanisms	Predicted, because of cross-system effects orchestrated at the level of the brain
View of “normal” biology	Based on statistical modeling, including population averages	None; all biological function is context-sensitive, and no context can be deemed “normal” in an absolute sense
Approach to therapeutics	Modification of specific mechanisms deemed to be “abnormal” or otherwise dysfunctional	Specific mechanisms may be modified or clamped to stabilize a highly disorganized system; full healing depends on facilitation of the brain’s endogenous orchestrative capacities, and modification of influences from the natural environment
View of free will and consciousness	Free will is a “user illusion” even in the healthy, since “consciousness” is an epiphenomenal product of determined, molecular processes	Free will and consciousness are “real” phenomena that have causal top-down efficacy for influencing lower levels of biology, even as they are correlated with molecular processes and may be compromised due to neural dysregulation
Derivation and substance of ethical considerations	Liberal political philosophy from the Enlightenment through the twentieth century generates ethical principles of autonomy, non-maleficence, beneficence, and justice; there is a need to avoid the naturalistic fallacy (“because it’s seen in nature, humans should do it too”)	Conventional biomedical ethics are upheld, even as new thinking in *neuroethics* presents the “augmented” principles of self-creativity, non-obsolescence, empowerment, and citizenship; neuroethics further integrates *new scientific insights* around brain function, with (potentially) *technology-aided advancements in brain function*; human freedom itself may be reconceptualized or experienced in new ways; there remains a need to avoid the naturalistic fallacy
Scientific approach	Linear models	Complexity, criticality, non-linear models

Sterling (2004) has contended that allostasis is necessary to *replace* the concept of homeostasis, which is itself flawed. In this view, homeostasis erroneously perceives that biological parameters maintain a categorically constant set point (they do not), and erroneously explains that these set points are maintained through corrective or reactive feedback (which is inconsistent with the ubiquity of anticipatory regulatory behaviors demonstrated throughout nature). It is the brain that acts as a data-integrating prediction-machine, to orchestrate operational set points and anticipatory behaviors in ways that support whole-organism health (with implications for species evolution). In the main, critics of Sterling’s original allostasis concept have focused on his point against biological constancy, and dismissed it as a mischaracterization of the thinking of Bernard and Cannon, who both recognized variability of biological functions. Consequently, there have been doubts as to whether the allostasis concept adds meaning beyond homeostasis (Dallman, 2003; Day, 2005). In contrast, there is little disagreement that the brain produces anticipatory behaviors, and increasingly researchers distinguish between “reactive homeostasis” and “predictive homeostasis” (e.g., Romero et al., 2009; Riede et al., 2017; Cardinali, 2018).

As stated from the outset of this essay, the PAO begins on the foundation of Sterling’s critique of the conceptions, data sets, typical modes of inference, and therapeutic concomitants (and limitations) of physiological research post-Cannon. Though Cannon made many statements which can be selectively highlighted to give the appearance that Sterling has misunderstood him (and Bernard’s appreciation for the brain has been unrecognized by many), a bird’s-eye view of homeostatic science makes clear that it is generally designed for the isolation of local factors, in hopes for discovery of “silver bullets”—with sparse attention to complex environments which will favor anticipatory variations in functional set points, or to the integrative and top-down role of the brain. The only way to validate the existence and efficacy of such bullets is to “factor out” the contributions of top-down neural regulation (e.g., the placebo effect) and environmental variations. Sterling can be forgiven for using Bernard and Cannon as straw-men in order to make a set of crucial points that have still yet to reach biomedical researchers as a community. There are vacuities in research which proceeds without a view informed by evolutionary processes (“the homeostasis model is essentially pre-Darwinian”; Sterling, 2012), the role of the brain as a prediction-machine, and consciousness itself.

Subsequent to Sterling, it is the McEwen view of allostasis (e.g., McEwen and Stellar, 1993; McEwen and Wingfield, 2003), that has been championed, gained currency, and been subject to its own criticism. In this formulation, homeostasis is defined as “stability of the physiological systems that maintain life,” and is taken to refer specifically to a few fundamental parameters including core temperature, pH, glucose level, and oxygen tension that are preserved within a relatively narrow range. Allostasis is defined as “stability through change,” or a process that *supports* homeostasis, and it encompasses a broader array of variations in activity patterns, for neuroendocrine hormones, cytokines, and other mediators, under conditions of acute stress. One critic of this “two-construct” perspective contends that it is founded on conceptual ambiguities, in that homeostasis should be conceptualized as a *state* (not a process), and that *processes which support* homeostasis can simply be labeled as “homeostatic mechanisms” (Day, 2005). A different critic has pointed out that alterations in hormonal secretions and other pathways are also fundamental to life (Dallman, 2003), such that a functional distinction between homeostasis and allostasis is untenable. Notably, the second criticism opens the door for Sterling’s more radical view—if all parameter maintenance in living systems can be traced to the support for life, then perhaps it is “homeostasis” that is rendered redundant. Moreover, parameter variabilities seem to be more a matter of degree than of kind. Acid-base balance, glucose, and oxygen tension also vary non-trivially depending on environmental factors including context-sensitive physical exertion, threat perception, altitude, nutritional status, and others. Even human body temperature appears to shift (if almost imperceptibly) in response to environmental factors (Cisse et al., 1991; Lui et al., 2014).

Nonetheless and conceivably, if the sciences which explore homeostasis—the collective methodologies essential to the maintenance and utility of a Kuhnian paradigm—were sufficiently broadened or revised, there would be no need to burden biological researchers with the additional concept of allostasis. *The necessity of “Sterling allostasis” (or the PAO) arises because data collection and causal inference in biology are substantially still driven by a methodology that systematically limits or ignores parameter alteration or variability that may be decisively influenced by factors from complex environments, as well as neural complexity including top-down efficacy for regulation of whole-body physiology (especially in higher vertebrates)*. Adequate updating of the classical view of homeostasis would entail the need for three formal revisions. Again, there would be a need for formal recognition of anticipatory behaviors (e.g., more recognition of “predictive” homeostasis). Second, there is a need for formal attention to top-down neural effects and their complexity (again considering that despite Cannon’s many studies of the brain, his *focus* was on the local “factors” of regulation, epitomized by the rubric of the wisdom of the *body*). Third, there is a need for an expansion in formal modeling of the complexity of the natural environment.

Thus, the PAO begins with two critical complexities that are foundations, not after-thoughts, and that are themselves interfaced through anticipatory regulation; and it avoids the possible introduction of “kludges” to rescue homeostasis. The PAO’s potential to resolve “reactive vs. predictive homeostasis” may be one illustration of its economy, in that it identifies “reactive homeostasis” as a kind of illusion. What *appears* to be a reactive (or “corrective”) process may be parsimoniously understood as another form of *anticipatory* behavior, if the complex environment has been “controlled” to appear monotonous and unchanging, and especially if neural regulation is relatively limited in its own complexity or criticality. An additional implication of conciliation is to offer lines of exploratory investigation for any given phenomenon that appears to be homeostatic. The PAO predicts that relative constancy in any biological parameter will yield to findings of increased variability if there are systematic alterations in either the complexity of neural dynamics (including the proximity to critical states), environmental complexity, or both. A straightforward illustration is to consider a subject who provides continuous daytime blood pressure readings for 10 consecutive days. He or she may show maintenance of an average (and “normal”) blood pressure in the vicinity of 120/80 for each day, irrespective of occasional changes from being seated to standing, or whether they read the morning newspaper or receive an animated neighbor as a guest. Consistency of the mean value at roughly 120/80 for each of those 10 days can be taken as empirical evidence for homeostasis. However if 10 days of readings were then to be collected from the same subject after they had begun a new role associated with high job strain (high demands with little autonomy, e.g., Landsbergis et al., 2013), or if new readings were collected under conditions of unusual variability of weather (e.g., high air mass, Morabito et al., 2008), then the data might contrast with the earlier experiment (that was “behaviorally and environmentally controlled”). The latter data sets might not suggest the homeostatic findings shown over the first 10 days, and they might show oscillations between alternative set points, consistent with propositions of the PAO. The crux of the investigational difference between the paradigms, is that if either of the second sets of findings were demonstrated with a *different subject*, the homeostatic paradigm would guide the researcher to *search for differences in their local factors* (i.e., differences in their genes or their expression, in today’s laboratories), whereas while the PAO would also be sensitive to genetic substrates, it would *search for differences in environmental and top-down neural regulatory influences*.

Following is a more provocative example of how homeostatic phenomena can be viewed as the special case of allostatic orchestrations that arise when a product of neural and environmental complexities is minimized. The constancy of core body temperature in homeotherms, despite major changes in seasonal weather, seems to give evidence for the McEwen-Wingfield formulation that homeostasis is the rule for a few fundamental parameters. Yet perhaps the narrow range is only a demonstration of relatively limited critical capacity for top-down neural regulation of temperature. Many mammal species reduce their core temperatures markedly during winter (i.e., they hibernate), at a time when their foraging opportunities are limited. Interestingly the evolutionary origins of hibernation are not genetically defined (Srere et al., 1992); there are different lineages that contain both hibernators and non-hibernators (suggesting that the trait has apparently been either gained or lost multiple times in evolution, without a “gene for hibernation”), pointing to differential patterns of gene expression or epigenetic processes (Morin and Storey, 2009). That humans do not hibernate can be interpreted as our own limitation in critical neural dynamics for temperature regulation, and the notion serves to remind that neural complexity should not be misunderstood to mean (or be conflated with) “cognitive intelligence for abstract or symbolic information processing.” With our species humility thus expressed, it also bears noting that human hibernation induction is being studied as a future therapy for preventing tissue ischemia (Lee, 2008) and also as a potential strategy for interplanetary space travel (Gemignani et al., 2015).

**Figure 5:**
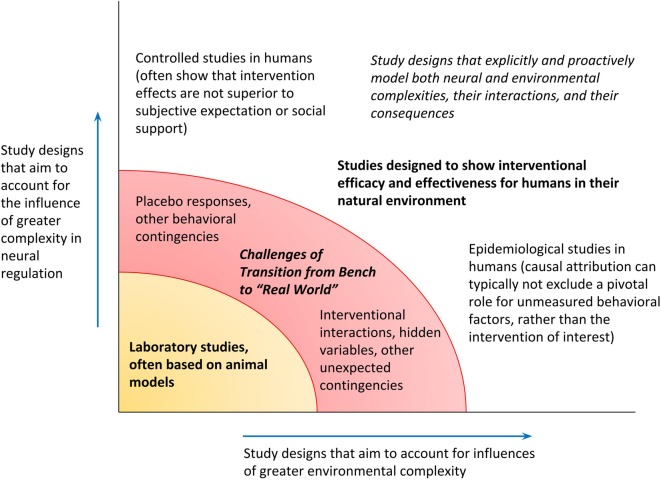
Research designs can be mapped along the degrees to which they admit complexity in the environment and top-down neural regulation. Confidence in the generalizability of findings from a given study, for applicability toward humans in their natural environment, depends on exceeding a threshold for recognizing both kinds of complexity (transition from the bench, to the “real” world).

To recapitulate, the PAO models biological operations in a way that expressly incorporates the complexity of the natural environment, the complexity of neural regulation as the organism’s “tool” for navigation within that environment, and anticipatory regulation as the process for interaction between the organism and its environment. As final granular examples of distinctions between the lines of research they motivate, the following are titles of hypothetical studies on glucose regulation. Elucidation of fine molecular factors may continue through a study such as, for example, “Aberrant expression of HMSTS disrupts glucose homeostasis through protease-mediated degradation of key receptor in obese mice.” Such studies may nonetheless still be more formally expressive about their limits and may do more to consider hidden variables in the environment or neural regulation, including animal handling patterns or even differences in the “lifestyles” of murine subjects. The PAO might inspire a report on “Stratification in frequency domain analysis of ecologically monitored glucose allostasis identifies new potential risk factors for pre-diabetes” (attention to context-dependence of physiological variability, focused on oscillatory properties of parameter estimates, for early disease prevention); or “Alteration in pre-prandial glucose allostasis through a comprehensive lifestyle intervention” (attention to anticipatory regulation and whole-person behavior); or “Allostatic intervention for critical brain dynamics in diabetics results in tighter glucose control without increasing episodes of hypoglycemia” (strategic promotion of agility for variation in allostatic states, for neurally-directed orchestrations of context-specific hormone levels). Figure 5 illustrates the mapping of studies along axes of variable attention to complexities in the natural environment and in top-down neural regulation and includes a category of research—studies that explicitly attend to complexities in both the environment and neural regulation—that as yet is largely nascent.

At this juncture it may also be helpful to identify socio-cultural and ethical currents as well as a key lacuna of scientific understanding—the physics of consciousness itself—which have likely contributed to the value and durability of the homeostatic paradigm, and may be presumed to associate with any application of the PAO (see also “Selected Considerations and Summary” section). Theological dictums have influenced progress in biology (for example in Descartes’ postulation of a dualistic reality that gave the church the province of the mind, and natural philosophers—scientists—the province of the body) no less than they have in physics. Separately yet relatedly, physicians and healers judiciously rely on the biological value of their patients’ subjective confidence. Bernard and Cannon formalized an experimental method explicitly designed to ascertain material causes and effects in living systems, without adducing idiosyncratic or sometimes exploitative ideas and practices of theologians, vitalists, physicians, or faith healers. These two giants of physiology are in a lineage that extends the legacy of the American polymath Benjamin Franklin one century and more before them, with respect to Franklin’s participation in a commission that falsified the “magnetism” claims of Franz Mesmer. It seems possible that Cannon’s own relative neglect of the brain’s top-down influences may have been partly motivated by his own principled ethical secularism (he was raised as a Unitarian Christian, and involved in numerous international political movements). Ethical secularism remains alive and well, easily recognizable in the commitments to liberal political philosophy dating back to the Western Enlightenment (e.g., the writings of John Locke, not modern partisan “liberalism”) which are central to modern biomedical ethics. Stated otherwise, modern biomedicine at its best may shun the benefits of the placebo response not out of antipathy to human self-empowerment, rather because it greater disdains the sense of charlatanism (or religious parochialism) that can sometimes be associated with its efficacious induction, and the distaste is exacerbated by the lack of a hold on the essential biology of subjective intentionality. The objectivity of the laboratory has not only identified the fine mediators of biological operations, it has also been constitutive for the needful expunging of “sin” in particular as an etiological construct in the understanding of health and disease.

Nonetheless and with appreciation for the weight of the aforementioned considerations, including the cost of progress and the work still undone, the thrust of the PAO is that the present need for advancements in neuroscience, healthcare, and beyond, points us to cease clinging to an era where the significance of subjectivity, or consciousness itself, is ignored in neural research or excluded with scrupulosity from the healthcare toolkit. To be clear, the PAO makes no call for a return to vitalistic reasoning, disease as “punishment,” or the methods of Mesmer. As the PAO admits consciousness and all its qualities, the “old” scientific conundrums and ethical dilemmas will not disappear; they will require advanced forms of conceptualization and engagement.

## Selected Considerations and Summary

### Implications of the Two Paradigms for Intervention on the Brain

Innovation is needed in the field of brain functional enhancement or advancement, that begins and ends with a homeostatic perspective. For example, a neuroprosthetic is a miracle if it permits recovery of homeostatic capacities for an individual with gross brain damage. Nonetheless even the most successful homeostatic interventions may entail systematic under-recognition of unintended consequences, because such effects may not be easily visible in the controlled context of early laboratory investigations. In contrast, the PAO perspective may be well suited to inform brain advancement objectives on larger scales, with heterogeneous populations, for objectives that may be less “controlling” yet potentially more ambitious. To clarify the differences entailed by homeostatic and PAO approaches to brain advancement, this section begins by considering how homeostasis and the PAO are associated with different approaches to “brain normality” and therapeutics in general.

Homeostasis begins with the (often unstated) presumption that there do exist “truly normal” forms of biological phenomena including brain functionality; that “true and discrete abnormalities” can be identified; and that the goal of therapeutics including brain-focused interventions is to “restore the state of normality.” While scientists acknowledge that the parameter estimates associated with these assumptions may be frail, there is little argument about the biological validity of the constructs themselves (normality, abnormality, and correction), and indeed without them, it is not clear how knowledge can be gained, or interventions can be devised. In contrast, the PAO definition of health as an active state of anticipatory behaviors with respect to the changing environment is not consistent with the identification of a single biological state that is objectively “normal.” An “average” specimen may be the one that is obliterated, in the next new environment; it may be the highly “unusual” individual that becomes the “next new normal” (Taleb, 2007, 2012). Moreover, free human beings make choices which produce compounding effects (in conjunction with differences in genetic expression and environmental exposures), eventuating in different states of brain, health, and life—which will continue to change over time. It is hardly obvious that universal criteria can exist for demarcating categorical thresholds of normality or pathology, in space *or* time.

Of note, homeostatic thinking may be the default mode of understanding even for some researchers who have embraced some concepts related to allostasis or allostatic load. Though it is possible to identify a wide array of mediators of top-down neural effects, it does not necessarily follow that intervention on one of those mediators represents a form of “allostatic therapy.” Rather it is attention to the brain’s unique and changing dynamics *per se*, to include its association with whole-organism behaviors in context, that is central to the Sterling principle of allostasis or the PAO. For example, many pharmacological strategies that are designed to clamp set points for neurotransmission, even if they “target allostatic load,” are still likely to entail the complications and limitations associated with homeostatic therapeutics (Sterling, 2004).

Broadly speaking, there are three general categories of interventional strategies that represent model solutions within the PAO. Beginning with the environmental context that first inspired his insights, Sterling (2004) has proposed that since social disruption is the ultimate cause for the brain to predict a need for elevated arousal, then it is repair of the social fabric that is ultimately necessary for the brain to revise its prediction and thereby re-orchestrate its downstream regulation. This category need not be limited to social factors (or political engagements) and can certainly include the built environment. Ulrich (1984) reported over 30 years ago that among patients hospitalized for cholecystectomy, those whose windows faced a natural setting had shorter stays than those who faced a brick wall, and today there are multi-stakeholder initiatives to imagine and build our surroundings in ways that are consistent with health promotion (Trowbridge et al., 2016). One hopes that architects and others may pay increasing attention to allostatic health not only in hospitals, also in urban planning and the design—and construction process—of educational structures, commercial and civic facilities, public housing, correctional institutions, and other locations where people spend hours, days, or years of their lives. Second and at the level of behaviors, the PAO points to the value of mental training, physical exercise, yoga, and related strategies, for their potential to support attenuation of elevated set points for the stress response system (e.g., Streeter et al., 2012; Pau et al., 2016). A third category entails a direct focus on the brain itself, for example through devices or other tools designed to support optimality of complex system dynamics (e.g., Lee et al., 2019) including the state of criticality (e.g., Ros et al., 2016).

Allostatic innovations across categories may be supported by imagination and new thinking from behavioral medicine specialists, mathematicians, primary care physicians, physicists, architects, urban planners, anthropologists, health system leaders, technology entrepreneurs, artists, epidemiologists, nutritionists, pharmacologists, yogis, self-empowerment gurus, and others. Whatever their character or provenance, allostatic strategies are likely to align with the non-linear sciences of complexity, and they may show that even subtle forms of well-placed intervention can have compounded consequences.

Brain-focused interventionalists should classify their approaches as being homeostatic or allostatic. Classification can occur by determining whether the objective is to “push” or clamp the brain toward a specific output parameter (e.g., alteration of mood, memory, performance metric, etc.), or to support the brain to move toward a pluripotent and flexible capacity for meeting the needs of changing situations. Allostatic intervention may still include the goal of “improvement” for a given output parameter, yet strictly speaking, such a change should be modeled as a secondary outcome (given its contingency on assumptions about the “better” which may or may not be durable). Whereas a homeostatic medication for sleep promotion may also cause lethargy that impairs attention, in contrast an allostatic intervention should support a state of “wholeness” that can be expressed as relaxation or concentration, depending on the contextual need. Moreover, robust allostatic interventions should have effects on bodily facets of the allostatic state, not only on cognitions, emotions or behaviors.

### Criticality as a Vehicle for Evolutionary Processes

Although the PAO arises from an evolutionary perspective, it does not adduce the ideas of adaptation or natural selection. Whereas a modern and molecular view of an adaptation conceives it as a genetic (hard-wired) variation, “field-tested” over repeated reproductive cycles that “judge” its fitness for a given environment (e.g., Dawkins, 1976), optimal anticipatory oscillation is the flexible, instantaneous, and iterated orchestration of multiple mechanisms across systems, to permit dynamic interaction with nature, when brain and environment are both changing at time cycles much faster than intergenerational gene transfer. Popper (1978) pointed out that many usages of *natural selection* are tautological—we know that some variants are more fit because they have survived; what permits some variants to survive, is being more fit. The PAO theorizes that the critical state of the brain (or critical states of proto-neural or neuro-analogous structures in organisms without brains), demonstrated behaviorally as optimality of anticipatory oscillation (or as “information-based fitness” within a general mathematical framework, e.g., Hidalgo et al., 2014), may serve as a general vehicle for evolutionary processes. Depending on the availability of genetic substrates and in association with other factors, the state of criticality may produce a variety of phenomena including differential reproductive success under conditions of resource scarcity (“natural selection”), sexual selection, altruism or cooperation, beauty *as such*, or other phenomena that are emergent or durable over evolutionary history.

### The PAO’s Philosophy of Freedom Preempts the Naturalistic Fallacy

Historically, early discussion of Darwinian evolutionary theory was dominated by attacks from theologians, who had held that creation of life on earth had happened once, perfectly, without subsequent changes of form. These antagonists did not easily accept the idea that humans could be physically related to members of the animal world. Though much antipathy to evolutionary perspectives may still be the product of ignorance, there is no question that there are some forms of “evolutionary thinking” that can also be problematic.

One error perpetuated by some evolutionists is the naturalistic fallacy (Moore, 1903). For present purposes, the idea is stated as follows: *Whatever the veridicality of scientific principles derived from observations about the earth’s natural biological past, these do not compel any form of reasoning or action with respect to the human present or future*. In simpler terms, the idea is no more complicated than the affirmation that, for example, to observe an alpha non-human primate maintain troupe stability, by killing a subordinate “naturally,” does not “justify” a human being’s decision to behave in a similar “natural” way. Or, because an evolutionary process associated with *struggle* has led to a certain state of outcomes that appear (to some) to have been desirable, it does not necessarily follow that *struggle* should be the guiding principle for producing new outcomes in the future. Consequences of the naturalistic fallacy are shown in darker chapters of modern history, post-*Origin*. Repressive movements such as eugenics, along with ideologies that informed twentieth-century fascism, for example, were products of misconceived justifications based on “survival of the fittest.”

Because allostasis explicitly recognizes the importance of the brain—and given that the human brain is associated with an advanced neocortex which supports higher-order information processing, to include ethical considerations—the PAO explicitly includes an engagement with questions that pertain to the nature of the good life. Elsewhere it has been contended that a new field of *allostatic neuro-education* can present many advantages for learners, especially by better preparing them for the rapidly changing environment of the twenty-first century (Gerdes et al., 2015). Allostatic neuro-education is guided by a philosophy of freedom (Steiner, 1894), which holds that the highest good is being free to live one’s life in a way guided by *ethical individualism*. Every new moment presents a new opportunity to make a new, context-sensitive choice. Ethical individualism requires the use of the moral imagination and the identification of options, which in turn depend on the development of a range of human capacities, the freedom to make choices among the options, the opportunity to experience the consequences of those choices, and to repeat. Being in its essence an approach to the brain which respects its wide-ranging and complex functionalities, the PAO has a natural alignment with the philosophy of freedom, and intuitively it seems plausible that the subjective feeling of freedom of choice may be the experiential dimension of the objectively defined state of criticality (Bettinger, 2017). The PAO further concords with principles of advanced neuroethics as recently articulated for the new era of self-transformative technologies (Shook and Giordano, 2016). The PAO is consonant with greater *self-creativity, non-obsolescence, empowerment*, and *citizenship*, which are intended as “upgrades” for the traditional concepts of autonomy, non-maleficence, beneficence, and justice, respectively.

The PAO thus preempts or otherwise rejects the naturalistic fallacy. No understanding of physical law can “force” the making of a particular human choice. And though there is much evidence to suggest that “freedom to choose” is a kind of fictive narrative that emerges in subjective consciousness only *after* neuronal processes and “decisions” have already occurred (e.g., Nørretranders, 1998), it is notable that studies have shown differences in the “neurological reality of choice” depending on whether a choice is, effectively, a “yes” or a “no” (Libet, 1999). Differences in the brain’s readiness potential have been shown depending on whether a subject has been induced to disbelieve in free will (Rigoni et al., 2011). There is much still to be learned in this area (Lavazza, 2016). In the interim, the PAO *perspective, as such*, has the potential to advance the science of brain advancement. The PAO illuminates a path to a wider range of choices, possibilities, and uses of the imagination, entailed by freedom; with respect to that freedom, it makes no prescriptions.

### A PAO Perspective on PTSD

As noted in Section “Progress and Limits in the Homeostatic Understanding of Posttraumatic Stress Disorder (PTSD)” there are anomalies that block the advance of understanding or practice with respect to PTSD, and many of them can be attributed to competition between foundational definitions held by different societal stakeholders including afflicted individuals and their loved ones, clinicians and healthcare advocates, military culture-bearers, clinical researchers, laboratory scientists, epidemiologists, and others. Whether these definitions are constructed upon, or against, the homeostatic paradigm (and either implicitly or explicitly), in either case, the persistence of homeostatic conceptualizations as our dominant “mental furniture” does not leave researchers or anyone else with much room for innovation at a fundamental level. Until there is a viable and attractive framework for biological research other than homeostasis, in some ways the limitations in scientific and societal progress around PTSD are predictable. If it has merit, the PAO should help to honor and integrate the most valuable elements of different perspectives that pertain to traumatic stress-related phenomenology, in a way that goes beyond the political brokering of ontological consensus, toward the construction of genuine and “needle-moving” solutions.

In the first place, a strong application of the PAO points us to dispense with the notion that the clinical construct of PTSD is a tenable way to distinguish between “healthy” and “diseased” individuals. As discussed in Section “Implications of the Two Paradigms for Intervention on the Brain,” it is problematic to define “normal” biology in any absolute sense, including for the state of the brain, and a visceral consciousness of this truth is appreciated greatly in, for example, communities of special operations military service members. To these individuals on mission, who have been trained to maintain extraordinary levels of alertness and sleep deprivation, it is counter-contextual to state that certain “symptom criteria”—including hypervigilance and sleep disturbance, core features of the clinical PTSD definition—can be used as indicators of their “abnormality.” Yet in a different setting, neither is it acceptable to suggest that these traits should have no negative impact on a service member’s ability to interact with civilians in a non-combat environment, or that service members should not receive tailored forms of healthcare or support from the governing agencies that trained them to develop their characteristics. If the “PTSD” concept must persist in order to serve civilian life, then perhaps it is a label that should simply be carried in a graded form by everyone, anywhere (and perhaps without the “D,” as some in the military prefer in order to minimize stigma, e.g., Blais and Renshaw, 2013). As a matter of self-insight if not clinical care, the PAO points us to consider that PTS is far more prevalent, or exists along a far more continuous spectrum than we currently model. Recognition of a continuum for a trait or functionality is thematic to the Research Domain Criteria framework of the National Institutes of Mental Health (Cuthbert and Insel, 2013), which aims to position behavioral health on a more robust scientific foundation than the descriptive and label-focused statistical manuals on which psychiatrists and psychologists now rely. Importantly, appreciation for a spectrum of states is not aimed to produce “disease creep” or the medicalization of everyday life, trends which can be dysfunctional when they are associated with the limitations and complications of homeostatic modeling and intervention. From an allostatic perspective and especially in the current era of accelerating changes (described as recurrent whiplash by even conservative commentators), the point is that our species will strain to thrive in the twenty-first century if we are not conscious of the character and the effects of acute and chronic stress, if we are not mature enough to acknowledge these stresses without blamesmanship, and if we lack efficacious, practical, economical, ethical, and genuinely *healthy* strategies for their prevention or management.

Second, not only is PTS (broadly construed) more endemic to modern life than homeostatic thinking might point us to consider (Selye, 1956), the PAO posits that we still have only a foggy appreciation for the pathways and consequences of stress effects, or the complexities which influence them. Chronic assaults on the brain are predicted to have impacts on all the subsystems which are under the regulation of central command, even if these influences originate from mental, social, cultural, or other “non-physical” sources. As noted in Section “Progress and Limits in the Homeostatic Understanding of PTSD,” it appears that non-physical factors such as cognitive appraisals may in some cases play a decisive role in the expression of behavioral pathology, raising the question that if free will is real, then what are its definable limits for producing top-down effects that recover “healthier” neural processes and their physical correlations? While the jury may still be out on this matter, meanwhile the multiple disturbances of subjective experience associated with clinical PTSD including negative cognitions, dysregulations of mood, and especially poor sleep, in conjunction with its risks for cardiovascular, metabolic, and other impairments such as age-related cognitive decline (e.g., Burri et al., 2013; Wang et al., 2016) should not be dismissed as probable statistical anomalies (and at an anecdotal level, the author of this article received an indelible imprint from an individual in her mid-50s with dementia, residing in an assisted living facility 15 years after being a responder to the 2001 terror attacks in New York City, where the author was also living at the time of the attacks; and see Clouston et al., 2016). Ramified effects from PTS are predicted by the PAO’s proposition that brain-body communication is the rule and not the exception. To further explore these relationships, it may be helpful for researchers to develop and apply new or existing tools for measuring the neural effects of relatively “subtle” environmental influences (including but not limited to “stressors”), alongside methodologies for measuring secondary effects on behavioral tendencies or physical organ function. (Notably, the general objective of defining and measuring “stress” is a persisting challenge, e.g., Koolhaas et al., 2011, and beyond the scope of the present article. A brief orientation, e.g., McEwen, 1998, reveals that allostatic load can associate with a variety of allostatic mediator profiles, not only elevation toward a flattened plateau, also a failure to adapt to repeated hits, or a blunted response sometimes associated with psychological burn-out. An effective special forces behavioral medic can discern without blood testing, when an operator has crossed over to being a biological “hot mess,” regardless of what that individual may say about their “stress”—and one wonders about the basis for such insight).

Third, the PAO predicts—and aims to inspire—new emphases and approaches to PTS(D) management and prevention, including allostatic interventional strategies described in Section “Implications of the Two Paradigms for Intervention on the Brain.” Given the complexities alluded to in PTS(D) ontology, pathogenesis, and comorbidity, it is unsurprising that there is no consensus regarding what to do about PTS(D) on a population basis, whether it can be prevented and if so then how, and whether efforts toward such challenges are worth the resources they might require. Though studies have been conducted on these questions (e.g., Cohen et al., 2017), it seems that the assumptions and data which would inform any “evidence-based” modeling of a *moonshot to prevent and eliminate the negative effects of stress*, for example, are likely to be unacceptably fragile or absent. Not only is the homeostatic evidence for subtle yet chronic stress effects inadequate for the kinds of conceptualizations entailed by the PAO, also the scientific appreciation for neural complexity is still only elementary (including the girders or limits of free will), and there is a need for advanced allostatic interventional strategy. Yet we should not be lulled into complacency excused by the lack of sufficient data collected from existing paradigms. *Homo sapiens* currently seem to be on path for exacerbations (or compounding) of stress effects that are already impairing our lives in joy and functionality (Harari, 2017). For reasons outside science, we may be at risk to traverse critical points of action for advancing human brain functional capacities—to include upgrades in our capacity to prevent and manage stress—before we will have established a conservative empirical consensus about how, precisely, to characterize or measure these stresses.

### Concluding Remarks and Summary

The reflections below aim to further exemplify the potential usefulness of the PAO with respect to the science of consciousness, as it pertains to the “placebo phenomenon,” and the science of human interactions with the global environment, in the present temporal context. Other implications of ongoing development and application of the two paradigms and their interaction with one another are beyond the scope of this essay.

There are data to indicate that effects from placebo interventions in clinical trials are increasing over time (e.g., Rief et al., 2009; Tuttle et al., 2015), and there is increasing study to understand placebo components and mechanisms (e.g., Schedlowski et al., 2015; Benedetti et al., 2016). While some interest stems from an allostatic orientation and aligns with a neuroethics of self-creativity or empowerment, some focus is strictly homeostatic, aimed to exploit the mediator molecules of placebo responding, or to eliminate individuals from clinical trials who appear to possess a genetic profile for such responding (in their “placebome”; Hall et al., 2015). While either increased homeostatic control of the placebo response—or increased refinement in its “out-factoring”—may support a variety of objectives, the latter agenda, especially, raises questions for both science and the neuroethics of non-obsolescence and citizenship. Is it a good idea, and ethical, for clinical trials to exclude individuals whose genetics may indicate that they are excellent placebo responders? For homeostatic science, such individuals dilute intervention-attributable effects and thereby increase sample size requirements. Yet placebo responders too pay taxes and healthcare insurance premiums, and as importantly, individuals whose consciousness is tuned for perception of their allostatic state and expression of its facets may show unanticipated outcomes that point toward alternative uses for an intervention. The orientation of the PAO is toward inclusion, in conjunction with a neuroethics that should lead to empowerment and new forms of insight, not fewer.

Geological data suggest that the earth is now in an Anthropocene epoch, characterized by human factor influences more than any other and that in recent decades we are experiencing a “Great Acceleration” (Lewis and Maslin, 2015). With increasing effects from climate change, technological disruptions including artificial intelligence and quantum computing and others, in conjunction with changing political, social, economic, and cultural currents, it seems probable that there are kinds and scales of changes that are still ahead of us. Yet if it is true that *Homo sapiens* are now the single largest influence on the natural environment as a complex whole, then it is no small matter to state that the essential function of the brain is to generate optimal anticipatory behaviors with *respect* to that self-same environment. The PAO predicts that if human brains can be successfully supported to be in allostatic states of criticality, with increased sensitivity to the environment and human impacts upon it, then they should demonstrate behaviors that anticipate the value of re-creating those environments so as to be recursively supportive of their allostatic criticality, the theorized bedrock of health itself. Allostatic agendas for brain advancement—to include both an allostatic *perspective* on the brain, as well as allostatic interventions—have the potential to generate virtuous cycles that leverage the inextricability of humans and their environments.

In the late nineteenth century, Claude Bernard laid the foundation for the homeostatic paradigm by showing that biological systems tend to maintain constancy, and Walter Cannon later used this idea to lead a long prolific run. As Bernard was concluding that the brain has a privileged position in its relationship to other organs, Charles Darwin brought forth a context-sensitive, evolutionary perspective to life on earth as a whole. Beginning 100 years later, Peter Sterling and Joseph Eyer re-realized the importance of environmental context; and they re-realized, with Bernard, that the brain is the upstream regulator of other organs. To synthesize those insights, Sterling and Eyer introduced the principle of *allostasis*, here expanded upon as the *PAO*, which posits that neurally directed anticipatory behavior—not corrective feedback—is the general principle of biological regulation. The PAO’s construct of the *allostatic state* serves to represent the integrated totality of brain-body interactions, and *optimal anticipatory oscillation*, potentially a function of the mathematically-defined state of criticality, is intended to spur the scientific imagination toward advancements in models, data, and interventions for positive health. As an evolutionary approach to brain advancement and in its alignment with a philosophy of freedom, the PAO aims to inspire new forms of allostatic thinking, research and action, including new attention toward currently under-attended classes of biological phenomena.

## Author Contributions

SL is responsible for the ideas presented in this manuscript, and was the sole writer.

## Conflict of Interest Statement

The author was formerly an employee at Brain State Technologies LLC, a company that has developed an allostatic neurotechnology, and he retains stock options with the company.

## References

[B1] Bai Z. Chang J. Chen C. Li P. Yang K. Chi I. (2015). Investigating the effect of transcendental medicine on blood pressure: a systematic review and meta-analysis. J. Hum. Hypertens. 29, 653–662. 10.1038/jhh.2015.6 25673114

[B2] Beggs J. M. Timme N. (2012). Being critical of criticality in the brain. Front. Physiol. 3:163. 10.3389/fphys.2012.00163 22701101 PMC3369250

[B3] Benedetti F. Carlino E. Piedimonte A. (2016). Increasing uncertainty in CNS clinical trials: the role of placebo, nocebo, and Hawthorne effects. Lancet Neurol. 15, 736–747. 10.1016/s1474-4422(16)00066-1 27106073

[B4] Benison S. Barger A. C. (2008). “Cannon, Walter Bradford,” in Complete Dictionary of Scientific Biography, (Vol. 15) eds Gillispie C. C. Holmes F. L. Koertge N. (Detroit: Charles Scribner’s Sons), 71–77.

[B5] Beristianos M. H. Yaffe K. Cohen B. Byers A. L. (2016). PTSD and risk of incident cardiovascular disease in aging veterans. Am. J. Geriatr. Psychiatry 24, 192–200. 10.1016/j.jagp.2014.12.003 25555625

[B6] Bernard C. (1878). Leçons sur Les Phénomènes de la vie Communs aux Animaux et aux Végétaux. Paris: Bailliere.

[B7] Bettinger J. S. (2017). Comparative approximations of criticality in a neural and quantum regime. Prog. Biophys. Mol. Biol. 131, 445–462. 10.1016/j.pbiomolbio.2017.09.007 29031703

[B8] Bevan A. T. Honour A. J. Stott F. H. (1969). Direct arterial pressure recording in unrestricted man. Clin. Sci. 36, 329–344. 5772109

[B9] Blais R. K. Renshaw K. D. (2013). Stigma and demographic correlates of help-seeking intentions in returning service members. J. Trauma. Stress 26, 77–85. 10.1002/jts.21772 23335155

[B10] Braam B. Huang X. Cupples W. A. Hamza S. M. (2017). Understanding the two faces of low-salt intake. Curr. Hypertens. Rep. 19:49. 10.1007/s11906-017-0744-z 28501983

[B11] Burke N. N. Finn D. P. McGuire B. E. Roche M. (2017). Psychological stress in early life as a predisposing factor for the development of chronic pain: clinical and preclinical evidence and neurobiological mechanisms. J. Neurosci. Res. 95, 1257–1270. 10.1002/jnr.23802 27402412

[B12] Burri A. Maercker A. Krammer S. Simmen-Janevska K. (2013). Childhood trauma and PTSD symptoms increase the risk of cognitive impairment in a sample of former indentured child laborers in old age. PLoS One 8:e57826. 10.1371/journal.pone.0057826 23469076 PMC3582641

[B13] Buysse D. J. (2014). Sleep health: can we define it? Does it matter? Sleep 37, 9–17. 10.5665/sleep.3298 24470692 PMC3902880

[B14] Buzsaki G. (2006). Rhythms of the Brain. New York, NY: Oxford University Press.

[B15] Cannon W. B. (1929). Organization for physiological homeostasis. Physiol. Rev. 9, 399–431. 10.1152/physrev.1929.9.3.399

[B16] Cardinali D. P. (2018). “The timed autonomic nervous system,” in Autonomic Nervous System: Basic and Clinical Aspects, (Cham, Switzerland: Springer), 19–56.

[B17] Carretero O. A. Oparil S. (2000). Essential hypertension: part I: definition and etiology. Circulation 101, 329–335. 10.1161/01.cir.101.3.329 10645931

[B18] Cisse F. Martineaud R. Martineaud J. P. (1991). Circadian cycles of central temperature in hot climate in man. Arch. Int. Physiol. Biochim. Biophys. 99, 155–159. 10.3109/13813459109146956 1713499

[B19] Clouston S. A. Kotov R. Pietrzak R. H. Luft B. J. Gonzalez A. Richards M.. (2016). Cognitive impairment among World Trade Center responders: long-term implications of re-experiencing the 9/11 terrorist attacks. Alzheimers Dement. 4, 67–75. 10.1016/j.dadm.2016.08.001 27626057 PMC5011166

[B20] Cocchi L. Gollo L. L. Zalesky A. Breakspear M. (2017). Criticality in the brain: a synthesis of neurobiology, models and cognition. Prog. Neurobiol. 158, 132–152. 10.1016/j.pneurobio.2017.07.002 28734836

[B21] Cohen G. H. Tamrakar S. Lowe S. Sampson L. Ettman C. Linas B.. (2017). Comparison of simulated treatment and cost-effectiveness of a stepped care case-finding intervention vs. usual care for posttraumatic stress disorder after a natural disaster. JAMA Psychiatry 74, 1251–1258. 10.1001/jamapsychiatry.2017.3037 28979968 PMC6583387

[B22] Conti F. (2002). Claude Bernard’s Des Fonctions du Cerveau: an ante litteram manifesto of the neurosciences? Nat. Rev. Neurosci. 3, 979–985. 10.1038/nrn985 12461555

[B201] Cooper S. J. (2008). From Claude Bernard to Walter Cannon. Emergence of the concept of homeostasis. Appetite 51, 419–427. 10.1016/j.appet.2008.06.005 18634840

[B23] Cuthbert B. N. Insel T. R. (2013). Toward the future of psychiatric diagnosis: the seven pillars of RDoC. BMC Med. 11:126. 10.1186/1741-7015-11-126 23672542 PMC3653747

[B24] Dallman M. F. (2003). Stress by any other name…? Horm. Behav. 43, 18–20. 10.1016/s0018-506x(02)00034-x 12614629

[B25] Darwin C. (1859). On the Origins of Species by Means of Natural Selection, or, the Preservation of the Favoured Races in the Struggle for Life. London: J. Murray.

[B26] Dawkins R. (1976). The Selfish Gene. Oxford: Oxford University Press.

[B27] Day T. A. (2005). Defining stress as a prelude to mapping its neurocircuitry: no help from allostasis. Prog. Neuropsychopharmacol. Biol. Psychiatry 29, 1195–1200. 10.1016/j.pnpbp.2005.08.005 16213079

[B28] Dekker J. M. Schouten E. G. Klootwijk P. Pool J. Swenne C. A. Kromhout D. (1997). Heart rate variability from short electrocardiographic recordings predicts mortality from all causes in middle-aged and elderly men: the Zutphen Study. Am. J. Epidemiol. 145, 899–908. 10.1093/oxfordjournals.aje.a009049 9149661

[B29] Dibner C. Schibler U. Albrecht U. (2010). The mammalian circadian timing system: organization and coordination of central and peripheral clocks. Annu. Rev. Physiol. 72, 517–549. 10.1146/annurev-physiol-021909-135821 20148687

[B30] DiNicolantonio J. J. Mehta V. O’Keefe J. H. (2017). Is salt a culprit or an innocent bystander in hypertension? A hypothesis challenging the ancient paradigm. Am. J. Med. 130, 893–899. 10.1016/j.amjmed.2017.03.011 28373112

[B31] Engel A. K. Fries P. Singer W. (2001). Dynamic predictions: oscillations and synchrony in top-down processing. Nat. Rev. Neurosci. 2, 704–716. 10.1038/35094565 11584308

[B32] Farr O. M. Ko B. J. Joung K. E. Zaichenko L. Usher N. Tsoukas M.. (2015). Posttraumatic stress disorder, alone or additively with early life adversity, is associated with obesity and cardiometabolic risk. Nutr. Metab. Cardiovasc. Dis. 25, 479–488. 10.1016/j.numecd.2015.01.007 25770759 PMC4404181

[B33] Fisher M. P. (2014). PTSD in the US military and the politics of prevalence. Soc. Sci. Med. 115, 1–9. 10.1016/j.socscimed.2014.05.051 24930003

[B34] Gemignani J. Gheysens T. Summerer L. (2015). “Beyond astronaut’s capabilities: the current state of the art,” in Proceedings of the 37th Annual International Conference of the IEEE Engineering in Medicine and Biology Society (EMBC) (New York, NY: IEEE), 3615–3618. 10.1109/EMBC.2015.7319175 26737075

[B35] Gerdes L. Tegeler C. H. Lee S. W. (2015). A groundwork for allostatic neuro-education. Front. Psychol. 6:1224. 10.3389/fpsyg.2015.01224 26347688 PMC4538224

[B36] Glaser R. Kiecolt-Glaser J. K. (2005). Stress-induced immune dysfunction: implications for health. Nat. Rev. Immunol. 5, 243–251. 10.1038/nri1571 15738954

[B37] Goldman L. (2015). Too Much of a Good Thing: How Four Key Survival Traits are Now Killing Us. New York, NY: Little, Brown and Company.

[B38] Graudal N. A. Hubeck-Graudal T. Jurgens G. (2017). Effects of low sodium diet versus high sodium diet on blood pressure, renin, aldosterone, catecholamines, cholesterol, and triglyceride. Cochrane Database Syst. Rev. 4:CD004022. 10.1002/14651858.CD004022.pub4 28391629 PMC6478144

[B39] Grmek M. D. (2008). “Bernard, Claude,” in Complete Dictionary of Scientific Biography, (Vol. 2) eds Gillispie C. C. Holmes F. L. Koertge N. (Detroit: Charles Scribner’s Sons), 24–34.

[B40] Hall K. T. Loscalzo J. Kaptchuk T. J. (2015). Genetics and the placebo effect: the placebome. Trends Mol. Med. 21, 285–294. 10.1016/j.molmed.2015.02.009 25883069 PMC4573548

[B41] Harari Y. (2017). Homo Deus: a Brief History of Tomorrow. New York, NY: Harper Collins.

[B42] Hidalgo J. Grilli J. Suweis S. Muñoz M. A. Banavar J. R. Maritan A. (2014). Information-based fitness and the emergence of criticality in living systems. Proc. Natl. Acad. Sci. U S A 111, 10095–10100. 10.1073/pnas.1319166111 24982145 PMC4104871

[B43] Johnson D. R. Lubin H. Rosenheck R. Fontana A. Sonthwick S. Charney D. (1997). The impact of the homecoming reception on the development of posttraumatic stress disorder: the west haven homecoming stress scale (WHHSS). J. Trauma. Stress 10, 259–277. 10.1002/jts.2490100207 9136091

[B44] Kalisch R. Müller M. B. Tüscher O. (2015). A conceptual framework for the neurobiological study of resilience. Behav. Brain Sci. 38:e92. 10.1017/S0140525X1400082X 25158686

[B45] Kasagi M. Huang Z. Narita K. Shitara H. Motegi T. Suzuki Y.. (2017). Association between scale-free brain dynamics and behavioral performance: functional MRI study in resting state and face processing task. Behav. Neurol. 2017:2824615. 10.1155/2017/2824615 29430081 PMC5752971

[B46] Kleiger R. E. Miller J. P. Bigger J. T. Jr. Moss A. J. (1987). Decreased heart rate variability and its association with increased mortality after acute myocardial infarction. Am. J. Cardiol. 59, 256–262. 10.1016/0002-9149(87)90795-8 3812275

[B47] Koob G. F. Le Moal M. (2001). Drug addiction, dysregulation of reward, and allostasis. Neuropsychopharmacology 24, 97–129. 10.1016/s0893-133x(00)00195-0 11120394

[B48] Koob G. F. Schulkin J. (2018). Addiction and stress: an allostatic view. Neurosci. Biobehav. Rev. [Epub ahead of print]. 10.1016/j.neubiorev.2018.09.008 30227143

[B49] Koolhaas J. M. Bartolomucci A. Buwalda B. D. de Boer S. F. Flügge G. Korte S. M.. (2011). Stress revisited: a critical evaluation of the stress concept. Neurosci. Biobehav. Rev. 35, 1291–1301. 10.1016/j.neubiorev.2011.02.003 21316391

[B50] Kox M. van Eijk L. T. Zwaag J. van den Wildenberg J. Sweep F. C. van der Hoeven J. G.. (2014). Voluntary activation of the sympathetic nervous system and attenuation of the innate immune response in humans. Proc. Natl. Acad. Sci. U S A 111, 7379–7384. 10.1073/pnas.1322174111 24799686 PMC4034215

[B51] Kuhn T. (1962). The Structure of Scientific Revolutions. Chicago: University of Chicago Press.

[B52] Landsbergis P. A. Dobson M. Koutsouras G. Schnall P. (2013). Job strain and ambulatory blood pressure: a meta-analysis and systematic review. Am. J. Public Health 103, e61–e71. 10.2105/ajph.2012.301153 23327240 PMC3673518

[B53] Lavazza A. (2016). Free will and neuroscience: from explaining freedom away to new ways of operationalizing and measuring it. Front. Hum. Neurosci. 10:262. 10.3389/fnhum.2016.00262 27313524 PMC4887467

[B54] Lee C. C. (2008). Is human hibernation possible? Annu. Rev. Med. 59, 177–186. 10.1146/annurev.med.59.061506.110403 18186703

[B56] Lee S. W. Gerdes L. Tegeler C. L. Shaltout H. A. Tegeler C. H. (2014). A bihemispheric autonomic model for traumatic stress effects on health and behavior. Front. Psychol. 5:843. 10.3389/fpsyg.2014.00843 25136325 PMC4118024

[B57] Lee S. W. Laurienti P. J. Burdette J. H. Tegeler C. L. Morgan A. R. Simpson S. L.. (2019). Functional brain network changes following use of an allostatic, closed-loop, acoustic stimulation neurotechnology for military-related traumatic stress. J. Neuroimaging 29, 70–78. 10.1111/jon.12571 30302866 PMC6586033

[B55] Lee P. Y. Yun A. J. Bazar K. A. (2004). Acute coronary syndromes and heart failure may reflect maladaptations of trauma physiology that was shaped during pre-modern evolution. Med. Hypotheses 62, 861–867. 10.1016/j.mehy.2004.02.004 15142637

[B58] Lewis S. L. Maslin M. A. (2015). Defining the anthropocene. Nature 519, 171–180. 10.1038/nature14258 25762280

[B59] Libet B. (1999). Do we have free will? J. Conscious. Stud. 6, 47–57.

[B60] Lui B. Cuddy J. S. Hailes W. S. Ruby B. C. (2014). Seasonal heat acclimatization in wildland firefighters. J. Therm. Biol. 45, 134–140. 10.1016/j.jtherbio.2014.08.009 25436962

[B61] Mayer E. A. Naliboff B. D. Chang L. Coutinho S. V. (2001). V. Stress and irritable bowel syndrome. Am. J. Physiol. Gastrointest. Liver Physiol. 280, G519–G524. 10.1152/ajpgi.2001.280.4.G519 11254476

[B62] McClintock M. K. Dale W. Laumann E. O. Waite L. (2016). Empirical redefinition of comprehensive health and well-being in the older adults of the United States. Proc. Natl. Acad. Sci. U S A 113, E3071–E3080. 10.1073/pnas.1514968113 27185911 PMC4896706

[B63] McEwen B. S. (1998). Protective and damaging effects of stress mediators. N. Engl. J. Med. 338, 171–179. 10.1056/NEJM199801153380307 9428819

[B64] McEwen B. S. Stellar E. (1993). Stress and the individual: mechanisms leading to disease. Arch. Intern. Med. 153, 2093–2101. 10.1001/archinte.1993.00410180039004 8379800

[B65] McEwen B. S. Wingfield J. C. (2003). The concept of allostasis in biology and biomedicine. Horm. Behav. 43, 2–15. 10.1016/s0018-506x(02)00024-7 12614627

[B66] McFarlane A. C. Lawrence-Wood E. Van Hooff M. Malhi G. S. Yehuda R. (2017). The need to take a staging approach to the biological mechanisms of PTSD and its treatment. Curr. Psychiatry Rep. 19:10. 10.1007/s11920-017-0761-2 28168596

[B67] Moore G. E. (1903). Principia Ethica. London: Cambridge University Press.

[B68] Morabito M. Crisci A. Orlandini S. Maracchi G. Gensini G. F. Modesti P. A. (2008). A synoptic approach to weather conditions discloses a relationship with ambulatory blood pressure in hypertensives. Am. J. Hypertens. 21, 748–752. 10.1038/ajh.2008.177 18443565

[B69] Morin P. Jr. Storey K. B. (2009). Mammalian hibernation: differential gene expression and novel application of epigenetic controls. Int. J. Dev. Biol. 53, 433–442. 10.1387/ijdb.082643pm 19412897

[B70] Najavits L. M. (2015). The problem of dropout from “gold standard” PTSD therapies. F1000Prime Rep. 7:43. 10.12703/p7-43 26097716 PMC4447050

[B71] Nørretranders T. (1998). The User Illusion: Cutting Consciousness Down to Size. New York, NY: Viking.

[B72] Pau M. Corona F. Pili R. Casula C. Sors F. Agostini T.. (2016). Effects of physical rehabilitation integrated with rhythmic auditory stimulation on spatio-temporal and kinematic parameters of gait in Parkinson’s disesase. Front. Neurol 7:126. 10.3389/fneur.2016.00126 27563296 PMC4980587

[B73] Pavlov V. A. Tracey K. J. (2015). Neural circuitry and immunity. Immunol. Res. 63, 38–57. 10.1007/s12026-015-8718-1 26512000 PMC4743890

[B74] Pickering T. G. Davidson K. Gerin W. Schwartz J. E. (2002). Masked hypertension. Hypertension 40, 795–796. 10.1161/01.HYP.0000038733.08436.98 12468559

[B75] Pickering T. G. Harshfield G. A. Kleinert H. D. Blank S. Laragh J. H. (1982). Blood pressure during normal daily activities, sleep, and exercise: comparison of values in normal and hypertensive subjects. JAMA 247, 992–996. 10.1001/jama.247.7.992 7057592

[B76] Pickering T. G. James G. D. Boddie C. Harshfield G. A. Blank S. Laragh J. H. (1988). How common is white coat hypertension? JAMA 259, 225–228. 10.1001/jama.259.2.225 3336140

[B77] Popper K. (1978). Natural selection and the emergence of mind. Dialectica 32, 339–355. 10.1111/j.1746-8361.1978.tb01321.x

[B78] Porges S. (2007). The polyvagal perspective. Biol. Psychol. 74, 116–143. 10.1016/j.biopsycho.2006.06.009 17049418 PMC1868418

[B79] Pruiksma K. E. Taylor D. J. Wachen J. S. Mintz J. Young-McCaughan S. Peterson A. L.. (2016). Residual sleep disturbances following PTSD treatment in active duty military personnel. Psychol. Trauma 8:697. 10.1037/tra0000150 27243567

[B80] Rees C. A. (2014). Lost among the trees? The autonomic nervous system and paediatrics. Arch. Dis. Child. 99, 552–562. 10.1136/archdischild-2012-301863 24573884

[B81] Riede S. J. van der Vinne V. Hut R. A. (2017). The flexible clock: predictive and reactive homeostasis, energy balance and the circadian regulation of sleep-wake timing. J. Exp. Biol. 220, 738–749. 10.1242/jeb.130757 28250173

[B82] Rief W. Nestoriuc Y. Weiss S. Welzel E. Barsky A. J. Hofmann S. G. (2009). Meta-analysis of the placebo response in antidepressant trials. J. Affect. Disord. 118, 1–8. 10.1016/j.jad.2009.01.029 19246102

[B83] Rigoni D. Kühn S. Sartori G. Brass M. (2011). Inducing disbelief in free will alters brain correlates of preconscious motor preparation: the brain minds whether we believe in free will or not. Psychol. Sci. 22, 613–618. 10.1177/0956797611405680 21515737

[B84] Romero L. M. Dickens M. J. Cyr N. E. (2009). The reactive scope model—a new model integrating homeostasis, allostasis, and stress. Horm. Behav. 55, 375–389. 10.1016/j.yhbeh.2008.12.009 19470371

[B85] Rooney K. L. Domar A. D. (2018). The relationship between stress and infertility. Dialogues Clin. Neurosci. 20, 41–47. 29946210 10.31887/DCNS.2018.20.1/klrooney PMC6016043

[B86] Ros T. Frewen P. Theberge J. Michela A. Kluetsch R. Mueller A.. (2016). Neurofeedback tunes scale-free dynamics in spontaneous brain activity. Cereb. Cortex 27, 4911–4922. 10.1093/cercor/bhw285 27620975

[B87] Rosenberg S. L. Miller G. E. Brehm J. M. Celedón J. C. (2014). Stress and asthma: novel insights on genetic, epigenetic, and immunologic mechanisms. J. Allergy Clin. Immunol. 134, 1009–1015. 10.1016/j.jaci.2014.07.005 25129683 PMC4252392

[B88] Sapolsky R. M. (2004). Why Zebras Don’t Get Ulcers. New York, NY: Henry Holt.

[B89] Schedlowski M. Enck P. Rief W. Bingel U. (2015). Neuro-bio-behavioral mechanisms of placebo and nocebo responses: implications for clinical trials and clinical practice. Pharmacol. Rev. 67, 697–730. 10.1124/pr.114.009423 26126649

[B90] Seeman T. Epel E. Gruenewald T. Karlamangla A. McEwen B. S. (2010). Socio-economic differentials in peripheral biology: cumulative allostatic load. Ann. N Y Acad. Sci. 1186, 223–239. 10.1111/j.1749-6632.2009.05341.x 20201875

[B91] Sega R. Cesana G. Bombelli M. Grassi G. Stella M. L. Zanchetti A.. (1998). Seasonal variations in home and ambulatory blood pressure in the PAMELA population. J. Hypertens. 16, 1585–1592. 10.1097/00004872-199816110-00004 9856358

[B92] Selye H. (1956). The Stress of Life. New York, NY: McGraw-Hill.

[B93] Shew W. L. Plenz D. (2013). The functional benefits of criticality in the cortex. Neuroscientist 19, 88–100. 10.1177/1073858412445487 22627091

[B94] Shook J. R. Giordano J. (2016). Neuroethics beyond normal: performance enablement and self-transformative technologies. Camb. Q. Healthc. Ethics 25, 121–140. 10.1017/S0963180115000377 26788953

[B95] Srere H. K. Wang L. C. Martin S. L. (1992). Central role for differential gene expression in mammalian hibernation. Proc. Natl. Acad. Sci. U S A 89, 7119–7123. 10.1073/pnas.89.15.7119 1379733 PMC49657

[B96] Steenkamp M. M. Litz B. T. Hoge C. W. Marmar C. R. (2015). Psychotherapy for military-related PTSD: a review of randomized clinical trials. JAMA 314, 489–500. 10.1001/jama.2015.8370 26241600

[B97] Steiner R. (1894). The Philosophy of Freedom: The Basis for a Modern World Conception. Trans. M. Wilson. East Sussex, UK: Rudolf Steiner Press.

[B98] Sterling P. (2004). “Principles of allostasis: optimal design, predictive regulation, pathophysiology and rational therapeutics,” in Allostasis, Homeostasis, and the Costs of Physiological Adaptation, ed. Schulkin J. (Cambridge, UK: Cambridge University Press), 17–64.

[B99] Sterling P. (2012). Allostasis: a model of predictive regulation. Physiol. Behav. 106, 5–15. 10.1016/j.physbeh.2011.06.004 21684297

[B100] Sterling P. (2014). Homeostasis vs. allostasis: implications for brain function and mental disorders. JAMA Psychiatry 71, 1192–1193. 10.1001/jamapsychiatry.2014.1043 25103620

[B101] Sterling P. Eyer J. (1988). “Allostasis: a new paradigm to explain arousal pathology,” in Handbook of Life Stress, Cognition and Health, eds Fisher S. Reason J. (New York, NY: J. Wiley and Sons), 629–649.

[B102] Streeter C. C. Gerbarg P. L. Saper R. B. Ciraulo D. A. Brown R. P. (2012). Effects of yoga on the autonomic nervous system, γ-aminobutyric-acid and allostasis in epilepsy, depression and post-traumatic stress disorder. Med. Hypotheses 78, 571–579. 10.1016/j.mehy.2012.01.021 22365651

[B103] Taleb N. (2007). The Black Swan: The Impact of the Highly Improbable. New York, NY: Random House.

[B104] Taleb N. (2012). Antifragile: Things That Gain from Disorder. New York, NY: Random House.

[B105] Tedeschi R. G. Calhoun L. G. (2004). Posttraumatic growth: conceptual foundations and empirical evidence. Psychol. Inq. 15, 1–18.

[B106] Thiel P. (2014). Zero to One: Notes on Startups, or How to Build the Future. New York, NY: Penguin Random House Company.

[B107] Trowbridge M. J. Worden K. Pyke C. (2016). Using green building as a model for making health promotion standard in the built environment. Health Aff. 35, 2062–2067. 10.1377/hlthaff.2016.1020 27834247

[B108] Tuttle A. H. Tohyama S. Ramsay T. Kimmelman J. Schweinhardt P. Bennett G. J.. (2015). Increasing placebo responses over time in US clinical trials of neuropathic pain. Pain 156, 2616–2626. 10.1097/j.pain.0000000000000333 26307858

[B109] Ulrich R. S. (1984). View through a window may influence recovery from surgery. Science 224, 420–421. 10.1126/science.6143402 6143402

[B110] VanElzakker M. B. Dahlgren M. K. Davis F. C. Dubois S. Shin L. M. (2014). From Pavlov to PTSD: the extinction of conditioned fear in rodents, humans and anxiety disorders. Neurobiol. Learn. Mem. 113, 3–18. 10.1016/j.nlm.2013.11.014 24321650 PMC4156287

[B111] Wang T. Y. Wei H. T. Liou Y. J. Su T. P. Bai Y. M. Tsai S. J.. (2016). Risk for developing dementia among patients with posttraumatic stress disorder: a nationwide longitudinal study. J. Affect. Disord. 205, 306–310. 10.1016/j.jad.2016.08.013 27552595

[B112] Williamson J. B. Porges E. C. Lamb D. G. Porges S. W. (2015). Maladaptive autonomic regulation in PTSD accelerates physiological aging. Front. Psychol. 5:1571. 10.3389/fpsyg.2014.01571 25653631 PMC4300857

[B113] Wolff J. L. Starfield B. Anderson G. (2002). Prevalence, expenditures and complications of multiple chronic conditions in the elderly. Arch. Intern. Med. 162, 2269–2276. 10.1001/archinte.162.20.2269 12418941

[B114] World Health Organization (1948). Preamble to the constitution of the world health organization as adopted by the International Health Conference, New York, 19–22 June, 1946; signed on 22 July 1946 by the representatives of 61 States (Official Records of the World Health Organization, no. 2, p. 100) and entered into force on 7 April 1948.

[B115] Yehuda R. Hoge C. W. McFarlane A. C. Vermetten E. Lanius R. A. Nievergelt C. M. (2015). Post-traumatic stress disorder. Nat. Rev. Dis. Primers 1:15057 10.1038/nrdp.2015.57 27189040

[B116] Yun A. J. Lee P. Y. Bazar K. A. (2004). Many diseases may reflect dysfunctions of autonomic balance attributable to evolutionary displacement. Med. Hypotheses 62, 847–851. 10.1016/j.mehy.2004.02.006 15142634

